# Convergence of distinct functional networks supporting naming and semantic recognition in the left inferior frontal gyrus

**DOI:** 10.1002/hbm.24953

**Published:** 2020-02-17

**Authors:** Zhansheng Xu, Bo Shen, Wael Taji, Pei Sun, Yuji Naya

**Affiliations:** ^1^ School of Psychological and Cognitive Sciences Peking University Beijing China; ^2^ Department of Psychology Zhejiang Normal University Jinhua China; ^3^ Yenching Academy Peking University Beijing China; ^4^ Department of Psychology, School of Social Sciences Tsinghua University Beijing China; ^5^ Tsinghua Laboratory for Brain and Intelligence Tsinghua University Beijing China; ^6^ Center for Life Sciences Peking University Beijing China; ^7^ IDG/McGovern Institute for Brain Research at Peking University Beijing China; ^8^ Beijing Key Laboratory of Behavior and Mental Health Peking University Beijing China

**Keywords:** declarative memory, familiarity, functional connectivity, inferior frontal gyrus, memory retrieval, naming, semantic control

## Abstract

Naming individual objects is accompanied with semantic recognition. Previous studies examined brain‐networks responsible for these operations individually. However, it remains unclear how these brain‐networks are related. To address this problem, we examined the brain‐networks during a novel object‐naming task, requiring participants to name animals in photographs at a specific‐level (e.g., “pigeon”). When the participants could not remember specific names, they answered basic names (e.g., “bird”). After fMRI scanning during the object‐naming task, the participants rated familiarity of the animals based on their sense of knowing. Since participants tend to remember specific names for familiar objects compared with unfamiliar objects, a typical issue in an object‐naming task is an internal covariance between the naming and familiarity levels. We removed this confounding factor by adjusting the familiarity/naming level of stimuli, and demonstrated distinct brain regions related to the two operations. Among them, the left inferior frontal gyrus triangularis (IFGtri) contained object‐naming and semantic‐recognition related areas in its anterior‐ventral and posterior‐dorsal parts, respectively. Psychophysiological interaction analyses suggested that both parts show connectivity with the brain regions related to object‐naming. By examining the connectivity under control tasks requiring nonlexical semantic retrieval (e.g., animal's body color), we found that both IFGtri parts altered their targeting brain areas according to the required memory attributes, while only the posterior‐dorsal part connected the brain regions related to semantic recognition. Together, the semantic recognition may be processed by distinct brain network from those for voluntary semantic retrievals including object‐naming although all these networks are mediated by the posterior‐dorsal IFGtri.

## Graphical abstract

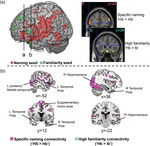

## 1 INTRODUCTION

Naming individual objects is a composite cognitive process and contains three stages after perception: (a) the semantic stage, where the item is recognized, and the semantic information is retrieved; (b) the lexical retrieval stage where the item's specific name is recalled; (c) the phonological retrieval stage where the phonemes of the retrieved name is recalled (Dell, 1986; Dell & O'Seaghdha, 1992; Dell, Schwartz, Martin, Saffran, & Gagnon, 1997; Foygel & Dell, 2000; Schwartz, Dell, Martin, Gahl, & Sobel, 2006). Substantial evidence from neuropsychological studies suggests that the semantic recognition should be distinguishable from the following lexical and phonological retrieval at the cognitive and neural level (Bi et al., 2011; Damasio, Grabowski, Tranel, Hichwa, & Damasio, 1996; Damasio, Tranel, Grabowski, Adolphs, & Damasio, 2004; Drane et al., 2013). Specifically, several studies on patients with lesions in the left anterior temporal lobe (ATL) revealed that patients showed intact object recognition, yet had name retrieval deficits (Bi et al., 2011; Damasio et al., 1996). Furthermore, another study revealed that right temporal lobe epilepsy (TLE) patients exhibited deficits in face recognition of famous persons without any problems in naming (Drane et al., 2013). Yet despite the suggested dissociation of the responsible brain regions for the sub‐functions, functional neuroimaging studies using cognitively normal participants have shown a large overlap between the brain regions related to semantic processing and those to lexical access including both lexical and phonological retrievals. For instance, some neuroimaging studies reported that the left ATL is involved in naming unique entities such as famous persons (Abel et al., 2015; Damasio et al., 1996), while other neuroimaging studies suggested its involvement in identification of famous persons or buildings (Gorno‐Tempini & Price, 2001; Olson, McCoy, Klobusicky, & Ross, 2013). In addition to the left ATL, the left pars triangularis of inferior temporal gyrus (IFGtri) was reportedly involved in both lexical retrieval task that required participants to retrieve names of animals, tools, and persons (Grabowski, Damasio, & Damasio, 1998) and postretrieval selection of semantic memory (Badre, Poldrack, Paré‐Blagoev, Insler, & Wagner, 2005; Badre & Wagner, 2007; Thompson‐Schill, D'Esposito, Aguirre, & Farah, 1997). Considering the inconsistent findings in the ATL between the neuropsychological studies and the neuroimaging studies, it remains unclear whether the overlapped brain regions such as the left IFGtri contribute to only one stage of the cognitive functions or all the stages during naming objects.

A possible explanation for the large overlap between the brain regions related to semantic processing and those to lexical access in the neuroimaging studies is that previous studies targeted only one profile of these cognitive functions and did not control an effect of the other (Damasio et al., 1996, 2004; Grabowski et al., 1998; Abel et al., 2015, for naming; Gorno‐Tempini & Price, 2001; Nakamura et al., 2001, for recognition). Since participants tend to retrieve specific names for familiar objects more than unfamiliar ones, there would be an internal covariance between the naming and familiarity levels of the object stimuli in those studies. Considering the lexical retrieval requires a semantic recognition as a precondition as well as a familiar object may remind participants of its specific name regardless of the task demand, the confounding effect embedded in the object stimuli might cause the largely overlapped brain regions across the previous neuroimaging studies.

**Figure 1:**
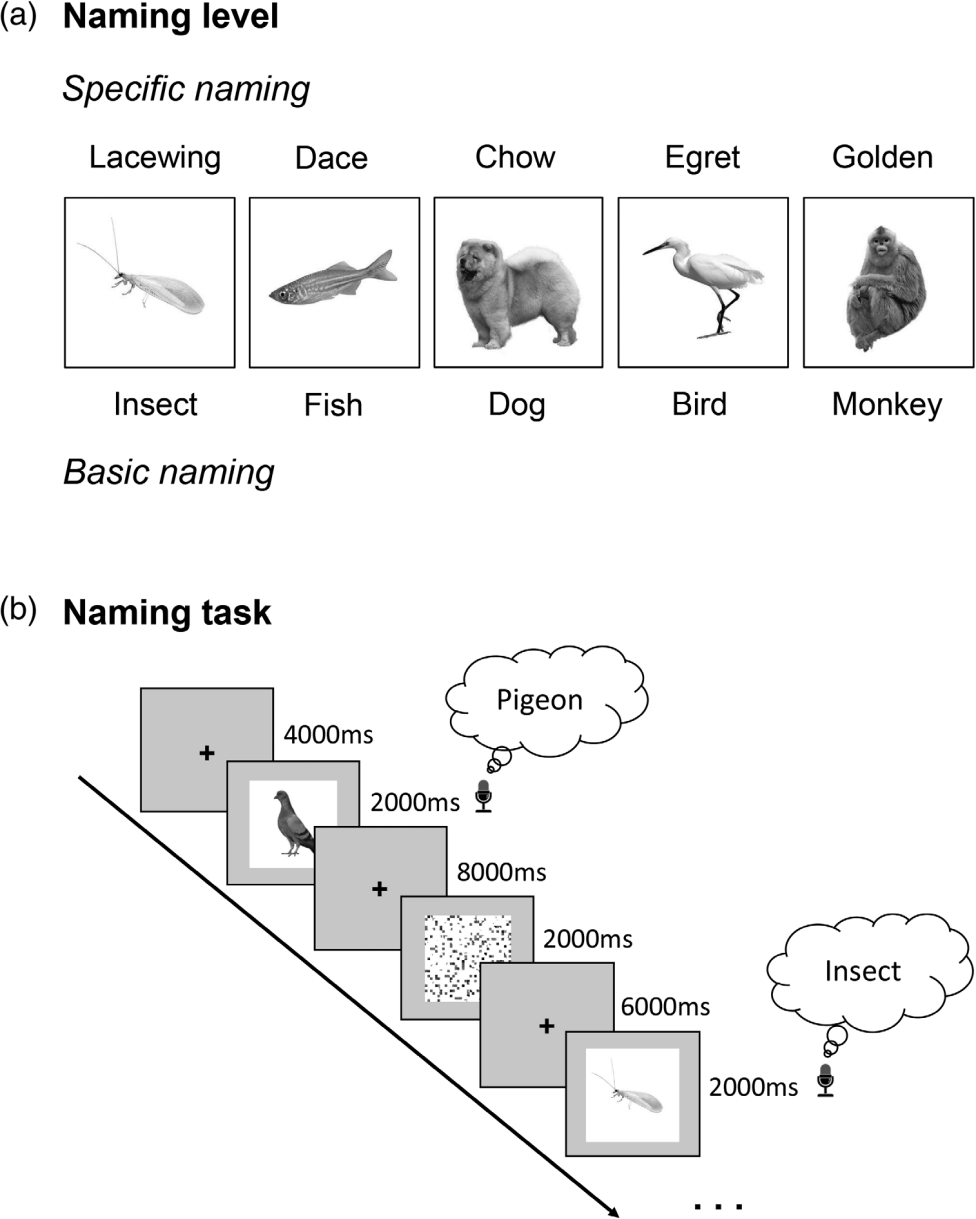
Overview of the behavioral paradigm. (a) Examples of stimuli used in both naming and feature‐retrieval tasks. Stimulus set consisted of five animal categories: 34 birds, 19 fishes, 27 insects, 23 dogs, and 17 monkeys. Each animal had two levels of naming: specific (e.g., pigeon) and basic (e.g., bird). (b) Schematic depiction of naming task. Participants tried to name the animals overtly at specific level or at the basic level when they could not remember the specific names of the animals

In the present study, we investigated neural substrates related with the individual cognitive processes for naming objects by measuring blood‐oxygenation‐level‐dependent (BOLD) fMRI signals during a newly devised objects‐naming task that required participants to overtly name the animals at the specific (subordinate) level as much as possible (Figure 1). In case they were unable to give the specific‐level name; they overtly answered the basic (superordinate) level name. The contrast between the specific naming and the basic naming was anticipated to detect brain activity related to the entire object‐naming process because the specific naming (e.g., “pigeon”) generally requires heavier mental load than the basic‐naming (e.g., “bird”) for its semantic (Hodges, Graham, & Patterson, 1995; Jefferies & Lambon Ralph, 2006), lexical (Howard, Nickels, Coltheart, & Cole‐Virtue, 2006) and phonological sub‐processes (Graves, Dell, Grabowski, Mehta, & Cupta, 2008; Rosch, Mervis, Gray, Johnson, & Boyes‐Braem, 1976). In the postscan test, the participants rated how strongly they felt they knew the objects in order to gauge their familiarity with the object (Belfi & Tranel, 2014; Kikyo, Ohki, & Miyashita, 2002). The participants tended to name familiar objects at specific level compared with unfamiliar objects. However, some object stimuli provided the participants with sufficient familiarity even when they answered basic names of the objects. The present experimental design thus allowed us to control the familiarity level difference (naming level difference) of objects when we tested the specific‐naming effect (high‐familiarity effect) on neural activity.

We predicted that brain regions associated with specific‐naming and high‐familiarity effects would be mostly separated under the control of the confounding effect between them. The specific‐naming effect would be observed in brain regions related to the lexical‐retrieval stage (e.g., the left ATL) and the phonological‐retrieval stage (e.g., pSTG). In addition to the representational areas, the specific‐naming effect may be also found in semantic control area, which shapes retrieval to suit a current situation or task demand (Lambon Ralph, Jefferies, Patterson, & Rogers, 2017). We hypothesized that the semantic control area would be also related with the semantic stage, and the individual networks related with the three stages might interact within the semantic control area when the participants named individual objects. In the present study, we found both specific‐naming and high‐familiarity effect in the left IFGtri, which is reportedly involved in selection of specific knowledge in line with an externally‐specified goal (Badre et al., 2005; Badre & Wagner, 2007; Davey et al., 2016). Previous neuroimaging studies examined the functional connectivity between the IFGtri and semantic representational regions, particularly a representational “hub” (e.g., the ATL) using the resting‐state paradigm (Hurley, Bonakdarpour, Wang, & Mesulam, 2015; Jackson, Hoffman, Pobric, & Lambon Ralph, 2016) or semantic judgment task in which subjects matched a probe word (e.g., “hen”) to the most semantically related target that was either strongly associated (e.g., “cage”) or conceptually similar (e.g., “robin”) to the probe (Jackson et al., 2016). The semantic judgment tasks required the global semantic associations between semantically related objects which would be represented in the amodal systems such as the ATL and posterior middle temporal gyrus (pMTG). However, it is still unsolved how the left IFGtri controls modal‐specific representational systems related to the word generation during a voluntary recall of individual object names which was tested in the present study.

To address this problem, we conducted psychophysiological interaction (PPI) analyses (Friston, 1994; Friston et al., 1997; O'Reilly, Woolrich, Behrens, Smith, & Johansen‐Berg, 2012), and compared the functional connectivity pattern between the left IFGtri and the representational areas for the to‐be‐retrieved modal‐specific contents during the naming task with those during control tasks requiring nonlexical semantic recollections (e.g., color of animals). Considering the role in the selection of specific knowledge (Badre et al., 2005; Badre & Wagner, 2007; Davey et al., 2016), we predicted the left IFGtri changes its connectivity pattern to the individual modal‐specific representational systems according to the to‐be‐retrieved contents required in the tasks. Results in the present study would clarify a contribution of the left IFGtri to the object‐naming process which requires interactions among distinct networks involved in the semantic, lexical retrieval, and phonological stages.

## 2 MATERIALS AND METHODS

### 2.1 Participants

The present study recruited 38 participants from Peking University (17 females, 21 males, mean age 22.7 ± 2.47 years). All 38 participants finished the naming task. No participants moved their heads during the task beyond a threshold (maximum translation of head motion <3 mm; Table S1). The threshold was based on previous literature (Jackson et al., 2016; Yu, Hu, Hu, & Zhou, 2014). All participants were native Chinese speakers and right‐handed, with normal or corrected to normal vision. No participants suffered from psychiatric or neurological disorders, had history of head injuries, or were on any psychoactive medication. A written informed consent form approved by the Institutional review board of the School of Psychological and Cognitive Sciences of Peking University was obtained from all the participants.

### 2.2 Stimuli

The stimulus set consisted of 120 black‐and‐white photographs of animals (see Figure S1), which were originally downloaded as colored photographs from the ImageNet website (Stanford Vision Lab, Stanford University). The animals of the stimulus set were separated into five basic categories: 34 birds, 19 fishes, 27 insects, 23 dogs, and 17 monkeys. The original photographs were subsequently resized to 350 × 350 pixels; color and background were removed. The visual stimuli were presented using the Psychtoolbox 3 package (Brainard, 1997) in MATLAB (MathWorks, Natick, MA).

### 2.3 Task design and procedure

#### 2.3.1 Main experiment (naming task)

**Figure 2:**
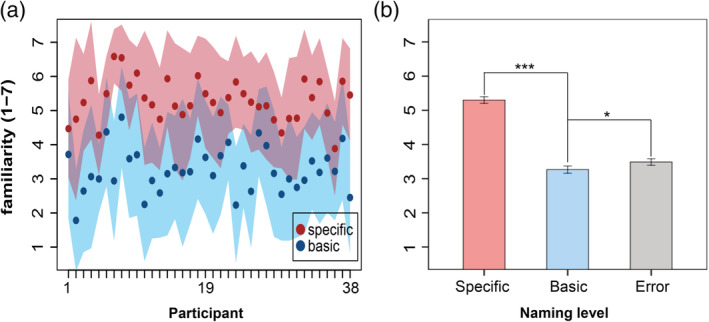
Familiarity ratings of specific and basic naming trials. (a) Single participant's familiarity ratings. Each dot indicates an average of familiarity rating of an individual participant across trials of each naming type (blue: basic; red: specific). SD is shown as shading around the mean (pink, specific naming; cyan, basic naming; purple, overlap between them). (b) Mean group familiarity ratings of the specific, basic, and error naming trials. Post hoc tests (Bonferroni correction) of the repeated measures ANOVA revealed that the mean familiarity rating of the specific naming trials was significantly higher than the basic‐naming trials (*p* < .001), and the familiarity rating of the error trials was also significantly higher than the basic trials (*p* = .035). Error bars indicate SEM. * *p* < .05, *** *p* < .001

Participants were instructed to overtly (in speech) name the animals presented on the screen at specific (subordinate) level (e.g., egret; Figure 1a). When unable to recall the specific animal name, they were required to name the animals at a basic level (e.g., bird). We informed the participants of the five basic level names (i.e., bird, fish, insect, dog, and monkey) before the scanning. To get enough number of trials for both specific and basic naming responses, we chose stimuli not only that would be easy for the participants to name at specific level, but also that would be difficult for the participants to remember the specific names in each animal category (Figure 2; see Figure S1). During the naming task, fMRI BOLD signals from the participants were measured and their vocal responses were recorded using an antimagnetic microphone system, which is equipped with a real‐time noise canceling function (FOMRI III, Optoacoustics Ltd, Or Yehuda, Israel).

In this naming task, each trial began with the appearance of a fixation point (“+”) on the center of the screen for 4–10 s (4, 6, 8, or 10 s), which was then replaced by a target animal picture for 2 s (Figure 1b). The participants were instructed to name the animal overtly during the period of picture presentation. In addition to the object naming, the task also included a low‐level baseline condition, under which a scrambled picture was presented for 2 s, and participants just needed to watch the picture passively. The naming task was conducted in two runs, each run consisting of 80 trials. The total time of each run was 12 min. The object presentation order for each run was pseudo‐randomized for each participant, with no consecutive trials presenting the same category (e.g., bird) of pictures. To reduce head motion of participants during vocalizing, a short induction briefing was given prior to fMRI scanning.

#### 2.3.2 Control experiments (feature‐retrieval task)

After the main experiment using the naming task, 36 participants finished one of the two control tasks both using feature‐retrieval tasks. Twenty participants (9 males, 11 females; mean age 23.1 ± 2.3 years) finished the color retrieval task. One participant was excluded from subsequent data analyses for the color‐retrieval task due to excessive head motion (> 3 mm maximum translation). Sixteen participants (11 males, 5 females; mean age 22.0 ± 2.4 years) finished the animal‐context retrieval task. One participant for excessive head motion (> 3 mm maximum translation), and two participants for misinterpretation of the instruction were excluded from subsequent data analyses for context‐retrieval task. In the feature‐retrieval tasks, participants were instructed to retrieve modal‐specific (i.e., color, context) contents of every animal vividly. We used the same animal photographs as in the naming task. Each trial of the feature‐retrieval task began with the appearance of a fixation point (“+”) in the center of the screen for 4–10 s (4, 6, 8, or 10 s), which was then replaced by a target animal picture for 2 s. During the presence of an animal picture, the participants were instructed to retrieve the animal's body color or context (habitat) silently, and then to press the left button if they retrieved successfully, or the right button if they could not retrieve. We chose pressing‐button response rather than vocal response for the feature‐retrieval task because the participants need longer time to describe body color or context compared with naming (e.g., some birds contain several colors), which may increase the head motion risk. In addition to the recollection, the task also included a low‐level control condition, under which a scrambled picture was shown for 2 s, and participants needed only to press the left button. The whole task included two runs, both consisting also of 80 trials. The total time of each run was 12 min.

#### 2.3.3 Postscanning test

After the fMRI scanning, we asked the participants to conduct a familiarity rating task. In this task, the participants were asked to evaluate the familiarity of the animal in each photograph on a scale of 1–7 (1 indicating extreme unfamiliarity, 7 indicating extreme familiarity), based on their sense of knowing rather than judging whether they felt that they had seen the photo during the scan.

### 2.4 Data acquisition and analysis

#### 2.4.1 fMRI data acquisition

Whole‐brain MRI data were collected using the 3T Siemens Prisma scanner at the Peking University MRI center. High‐resolution 3D structural images were acquired using the 3D‐MPRAGE sequence (TR, 2530 ms; TE, 2.98 ms; flip angle, 7°; matrix size, 448 × 512; voxel size 0.5 × 0.5 × 1 mm^3^). BOLD signal was acquired using a multiband echoplanar imaging (EPI) sequence (TR, 2000 ms; TE, 30 ms; flip angle, 90°; matrix size, 112 × 112; voxel size, 2 × 2 × 2 mm^3^, 64 slices with gap of 0.1 mm).

#### 2.4.2 fMRI data preprocessing

The fMRI data were preprocessed and analyzed using SPM12 (Wellcome Trust Centre for Neuroimaging, London, UK) and MATLAB software (MathWorks). Preprocessing of the functional MRI data included slice timing, realignment (head motion correction), co‐registration, segmentation, normalization, smoothing, and high‐pass filtering. Slice timing (sinc interpolation) was used to correct the differences in image acquisition time between slices within a TR. Subsequently, realignment (3D rigid‐body transformation) was conducted to correct head motion. Using this method, head motions in the naming task were effectively corrected (see Table S2). To evaluate if the motion artifacts are different between the naming task and feature retrieval tasks, we calculated frame‐wise displacements using following formula:

$${\mathrm{FD}}_{i}=|\Delta d_{\mathrm{ix}}|+|\Delta d_{\mathrm{iy}}|+|\Delta d_{\mathrm{iz}}|+|\Delta \alpha_{i}|+|\Delta \beta_{i}|+|\Delta \gamma_{i}|,\text{where}\;\Delta d_{\mathrm{ix}}=d_{\left(i-1\right)x}-d_{\mathrm{ix}},$$

and similarly for the other rigid body parameters [*d*_*ix*_ *d*_*iy*_ *d*_*iz*_ *α*_*i*_ *β*_*i*_ *γ*_*i*_] (Power, Barnes, Snyder, Schlaggar, & Petersen, 2012). The frame‐wise displacements were not significantly different between the naming task and the control tasks [*t* (68) *=* 0.637, *p* = .57], indicating that the overt responses during the naming task did not make larger motion artifacts than the button pressing which is commonly used in fMRI experiments. To normalize functional images, each participant's structural brain image was coregistered to the mean functional image and was subsequently segmented. The parameters obtained in segmentation were used to normalize each participant's functional image onto the Montreal Neurological Institute (MNI) space (resampling voxel size was 2 × 2 × 2 mm^3^). All volumes were spatially smoothed using an isotropic 6‐mm full‐width at half‐maximum Gaussian kernel. In addition, a high pass filtering was used to remove low‐frequency drifts.

#### 2.4.3 Data analysis

##### General linear model (GLM) analysis

For the naming task (main experiment), to detect the brain regions related to specific‐naming and recognition processing for familiar objects, a categorical general linear model (GLM) analysis of the functional MRI data was performed for whole brain in each participant. Because the effects of naming level and familiarity level were confounded in the stimuli (i.e., the specific‐naming items usually had higher familiarity ratings than the basic‐naming items), we examined these effects via a single categorical GLM analysis in order to control the confounding factor. Based on the participants' naming responses and familiarity ratings, naming task trials were divided into three conditions: the high familiarity and specific‐naming (*HS*) trials, the high familiarity and basic‐naming (*Hb*) trials, and low familiarity and basic‐naming (*lb*) trials. The “HS” trials were the specific‐naming trials of which familiarity ratings were above the mean value of individual participants. The “Hb” trials were the basic‐naming trials for which the familiarity ratings were above the mean value of individual participants. Then, we chose the “lb” trials from the basic‐naming trials with the lowest familiarity rating (e.g., 1) until the number of the “lb” trials neared that of the “Hb” trials. The mean trial numbers across the participants were 29.3 ± 8.3 (“HS”), 20.8 ± 8.1 (“Hb”), and 24.3 ± 6.9 (“lb” trials). Taken together, this GLM analysis included 5 main regressors: the effect of *HS* trials, the effect of *Hb* trials, the effect of *lb*, the effect of baseline condition trials, and the effect of other no‐interest trials. We did not incorporate the low familiarity and specific naming (lS) condition into the GLM, because there are not enough trials of the “lS” condition (the mean trial number was 3.6 ± 2.9). Furthermore, the six motion regressors were also included as nuisance regressors. For group‐level analysis, we entered the contrast images (e.g., “Hb > lb” contrast) that were generated by the subject‐level GLM analyses into a second‐level one‐sample *t*‐test.

In addition to the categorical GLM analysis, the familiarity effect was also examined using a parametric modulation analysis based on the familiarity ratings (seven levels) within the basic‐naming trials. In the parametric analysis, the polynomial functions up to the second order were used. This analysis included five main regressors: the effect of specific‐naming trials, the effect of basic‐naming trials, the effects of the first and second order of familiarity ratings within the basic‐naming trials, and the effect of other trials (including the error trials). In addition, the six motion regressors were also included as nuisance regressors. Subject‐level analyses were run to generate SPM contrast images, and these contrast images were entered into a group‐level random‐effects GLM.

For each of the control experiments (color‐retrieval and context‐retrieval tasks), a categorical GLM analysis was performed for whole brain in each participant. Each GLM analysis included four main regressors: the effect of successful‐retrieval (“Yes”) trials, the effect of unsuccessful‐retrieval (“No”) trials, the effect of baseline condition trials (i.e., scrambled‐picture trials), and the effect of other no‐interest trials (i.e., the error trials). In addition, the six motion regressors were also included as nuisance regressors. In group‐level analysis, we entered the contrast images (i.e., “Yes > No” contrast) that were generated by the subject‐level GLM analyses into a second‐level one‐sample *t*‐test

##### PPI analysis

To assess the functional connectivity patterns contributing to a particular cognitive function, whole brain PPI analyses were conducted by performing a separate GLM analysis involving three main regressors: (a) the “physiological” regressor; (b) the “psychological” regressor; and (c) the “PPI interaction” regressor (Friston, 1994; Friston et al., 1997; O'Reilly et al., 2012). In addition, the six motion regressors were also included as nuisance regressors for each session.

As the physiological regressor, activities of a spherical brain region within six millimeters radii (i.e., seeds) were used. In left IFGtri, we defined five seeds in total based on the categorical GLM analysis which evaluated the effects of *HS*, *Hb*, and *lb* trials together for the examinations of both naming (“*HS* > *Hb*”) and familiarity (“*Hb* > *lb*”) effects. The first four seeds (i.e., naming seed, familiarity seed, color seed, and context seed) were determined as peaks in the t values of the second‐level one sample *t*‐test which tested whether the results of GLM analyses were positive across the participants in each voxel within the left IFG‐tri (*p* < .05, voxel‐wise FDR correction). One more seed was chosen according to previous literature (Badre et al., 2005; Whitney, Kirk, O'Sullivan, Lambon Ralph, & Jefferies, 2011) because the familiarity seed, color seed, and context seed are all close to it, which we referred to as Badre's seed in the present study. By using the Badre's seed, the present findings can be directly compared with those of the previous studies. Positions of the naming seed and familiarity seed correspond to the peak positions of left IFGtri for the specific‐naming contrast and high‐familiarity contrast in Table 1, respectively. Positions of the color seed, context seed, and Badre's seed were shown in the Figure 5. To compare with the lateral prefrontal cortex (PFC) seeds (i.e., left IFGtri), we also defined a seed in medial PFC, which was chosen based on high‐familiarity contrast (“Hb > lb”). As the psychological regressor, trial types related with a particular functional effect were used. A total of four contrasts between trial types were examined for each brain seed: the specific‐naming (contrast: HS > Hb), the recognition of familiar objects (contrast: Hb > lb), and the two object‐feature retrievals (contrast: successful retrieval > unsuccessful retrieval). The interaction regressor was used to identify voxels in which functional activity covaried in a task‐dependent manner with the seed region (Friston, 1994; Friston et al., 1997). Subject‐level PPI analyses were run to generate SPM contrast images similar to a subject‐level GLM model and then these contrast images were entered into a group‐level random‐effects GLM.

**Table 1:** Brain regions associated with specific naming or high familiarity after controlling the confounding factors

				MNI coordinates		
Brain regions	Left/right	Cluster size (voxels)	*t* value (peak)	*X*	*Y*	*Z*
*Specific naming (HS > Hb)*						
Temporal pole	L	44	3.88	−48	20	−14
Temporal Sup^a^	L	1,150	7.28	−56	−18	12
Temporal Sup^a^	R	1,157	7.06	52	−2	−4
Frontal Inf Tri^a^	L	169	5.72	−52	44	−2
Frontal Inf Orb^a^	L	312	4.62	−42	18	−6
Frontal Sup Medial	L	12	3.17	−4	32	40
Temporal Mid	L	83	3.78	−58	−34	−16
Temporal Inf^a^	L	217	4.39	−52	−56	−10
Frontal Inf Oper	L	229	4.16	−52	12	10
Cingulum Ant	L/R	123	4.76	4	32	16
Cingulum Mid	L/R	229	4.79	6	8	34
Parietal Inf^a^	L	719	5.85	−54	−24	38
Supramarginal^a^	L	402	4.80	−46	−36	28
Supramarginal	R	60	4.91	48	−32	46
Precentral	L	82	3.82	−18	−24	62
Supp motor area	R	22	3.84	8	−2	60
*High familiarity (Hb* > *lb)*						
Frontal Inf Tri^a^	L	27	4.89	−52	26	14
Frontal Inf Orb^a^	L	66	5.15	−22	24	−14
Rectus^a^	L	126	5.89	−4	36	−16
Frontal Med Orb^a^	L/R	312	5.52	−4	50	−10
Frontal Sup Medial^a^	L/R	257	5.23	−14	60	18
Cingulum Ant^a^	L	48	4.31	0	50	6
Precuneus	L	10	4.23	−8	−56	28
Occipital Sup^a^	L	170	5.53	−10	−98	6

*Note*: Only clusters with a significant activity of voxel‐level threshold *p*_FDR‐corr_ < .05 are reported.

Abbreviations: L, left; R, right; and L/R, the clusters that covered bilateral hemispheres.

a The clusters go through the cluster‐wise FWE correction (*p*_FWE‐corr_ < .05 at cluster level).

##### ROI analysis of PPI effects

To compare the connectivity patterns among the modalities (i.e., word, color, and context) of memory contents for their retrieval, ROIs analyses were performed using the analysis results of the whole‐brain PPI described above. First, cubical ROIs (length of a side of the cube was 6 mm; Yu et al., 2014; Yu, Zhou, & Zhou, 2013; Zhang, Yu, Yin, & Zhou, 2016) were constructed around the peak coordinates from the whole‐brain PPI results in key brain regions identified from previous literature, including the SMA, pSTG, TP, hippocampus, fusiform gyrus (FG), and parahippocampal gyrus. Among these, the SMA, left TP, and left pSTG were related to lexical retrieval and speech process (Damasio et al., 1996; Hertrich, Dietrich, & Ackermann, 2016; Hickok, 2009); the right TP, and hippocampus were related to familiarity (Drane et al., 2013; Leveroni et al., 2000); the FG was related to color information retrieval (Simmons et al., 2007; Wang et al., 2013); and the parahippocampal gyrus was related to context retrieval (Davachi, 2006; Staresina, Duncan, & Davachi, 2011). Second, beta values of the interaction regressors (formed for each seed area under four contrasts: specific naming; high familiarity; color retrieval; and context retrieval) were calculated for each voxel inside individual ROIs, and then the beta values of all voxels in each ROI were averaged. Third, these subject‐level beta values were entered into a group‐level one‐sample *t*‐test (Bonferroni corrected).

## 3 RESULTS

### 3.1 Behavioral results

During the naming task, participants pronounced specific name and basic name correctly for 31.2 ± 9.0% and 59.1 ± 10.4% of the total trials and made errors in 9.7 ± 4.8% (mean ± SD, *n* = 120). After the fMRI scanning, we examined how strongly the participants felt they knew the animals presented in the naming task. In the postscan familiarity test, we found a significant difference in familiarity ratings among the animals with different naming levels [*F* (2, 74) *=* 214.3, *p <* .0001, repeated measures ANOVA]. Post hoc tests using the Bonferroni correction revealed that the participants rated a significantly higher familiarity for the specific‐named items than basic‐named items (*p <* .0001, two tails; Figure 2b). This pattern was consistent across all participants (Figure 2a). Behavioral results demonstrated that the familiarity ratings covaried with naming performance, that is, the specific‐named items usually had higher familiarity ratings than the basic‐named items. To tease apart the effect of naming from familiarity, we divided the trials of the naming task based on the participants' naming performances and familiarity ratings into three conditions: (1) “HS” trials, (2) “Hb” trials, and (3) “lb” trials (see Section 2.4.3). As a result, the familiarity level was adjusted between the “HS” trials (mean familiarity = 5.50 ± 0.37) and the “Hb” trials (mean familiarity = 5.44 ± 0.39; *p* = .123, two‐tailed *t*‐test), and their familiarity levels were substantially larger than that of the “lb” trials (mean familiarity = 1.53 ± 0.57, *p* < .0001 for both, Bonferroni corrected).

### 3.2 Brain regions showing naming effect and familiarity effect

**Figure 3:**
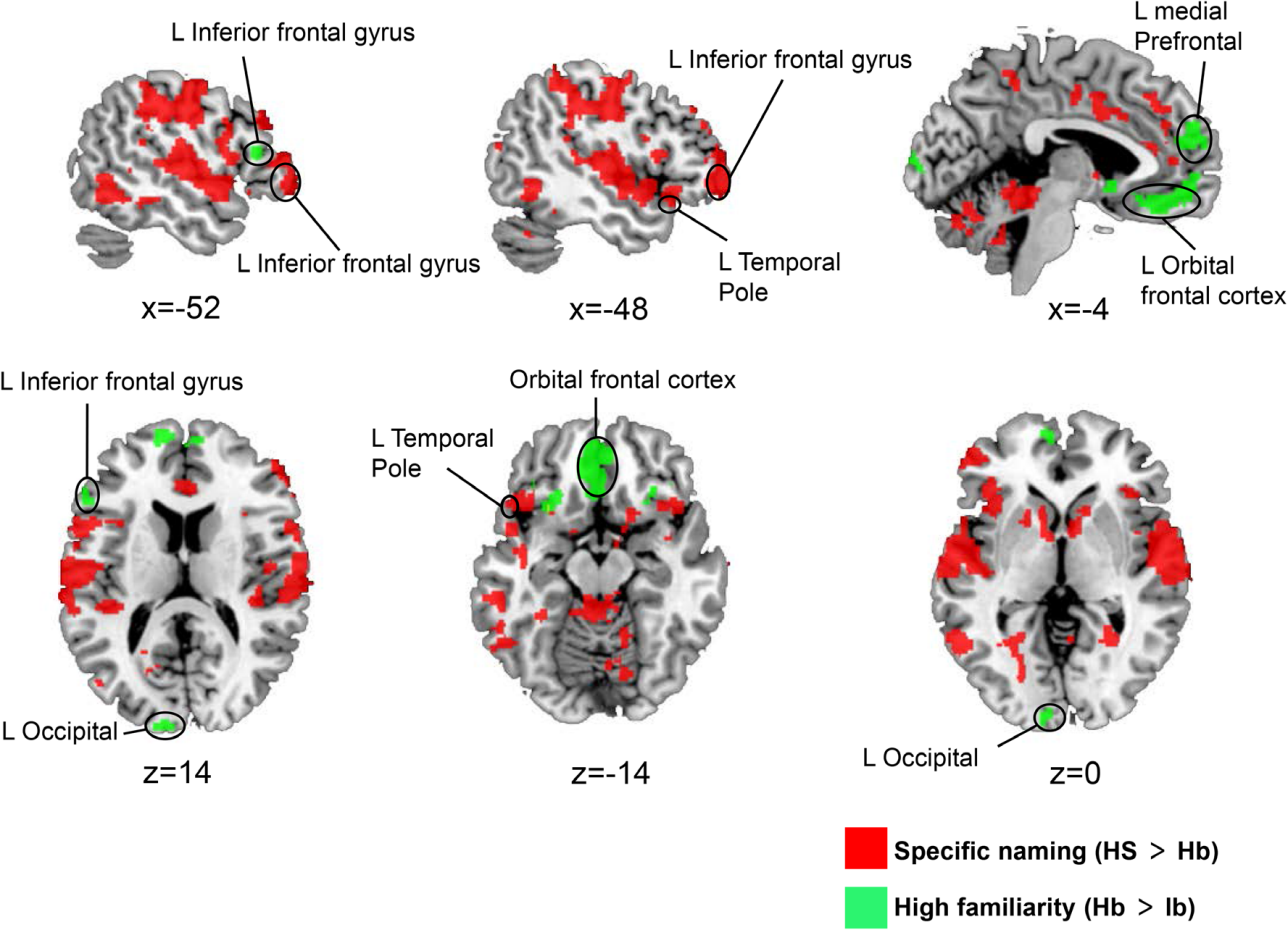
Brain regions associated with specific naming or high familiarity. Brain regions related to specific naming are shown in red, while those related to high familiarity are shown in green (*p* < .05, FDR corrected at voxel level). HS, high familiarity under the specific‐naming level; Hb, high familiarity under the basic‐naming level; lb, low familiarity under the basic‐naming level

Using the three trial‐conditions (“HS”, “Hb”, and “lb”) as regressors, we successfully differentiated the brain regions that responded to specific naming effect (i.e., “HS > Hb” contrast) from the brain regions that responded to high familiarity effect (i.e., “Hb > lb” contrast; Table 1, Figure 3; the statistical threshold of the group‐level analysis is *p* < .05, FDR corrected at voxel level). This result contrasts with the large overlap between the brain regions associated with the two effects when we examined each effect irrespective of the other (see Supplemental Data Analysis and Figure S3). Considering the internal covariance between the familiarity ratings and the naming performances of the stimuli (Figure 2b), the overlap brain regions between the two effects may be due to their confounding effect which were present in the stimuli. The brain regions responsible for specific naming included the left temporal pole (TP, BA38), bilateral superior temporal gyrus (STG, BA41), bilateral supramarginal gyrus (BA40), and left pMTG (BA21) (*p* < .05, FDR corrected at voxel level, Table 1). Meanwhile, the brain regions associated with familiarity indexing included the bilateral medial PFC (medial parts of BA9/10), bilateral OFC (BA11), and bilateral occipital cortex (BA18/19) (*p* < .05, FDR corrected at voxel level, Table 1). The results of familiarity effect from categorical GLM contrast (“Hb > lb”) was confirmed by a parametric modulation analysis with familiarity ratings (1–7) as the modulation parameter. While the two analyses showed a similar pattern of brain activation, familiarity effect in the right TP (BA38), right hippocampus, and bilateral calcarine (BA30) reached a statistical significance only in the parametric modulation analysis (see Table S3), presumably because of its statistical advantage compared with the categorical contrast.

In addition to the specific brain regions responsible for the specific naming or high familiarity, we found the left IFGtri (BA45) to be a commonly activated brain region although the activation sites differed between the two effects within it (Table 1 and Figure 3). Our results suggest that specific‐naming and processing for familiar objects during object identification are supported by distinct brain networks, which may be linked in the left frontal lobe.

### 3.3 Connectivity patterns for specific naming and high familiarity

**Figure 4:**
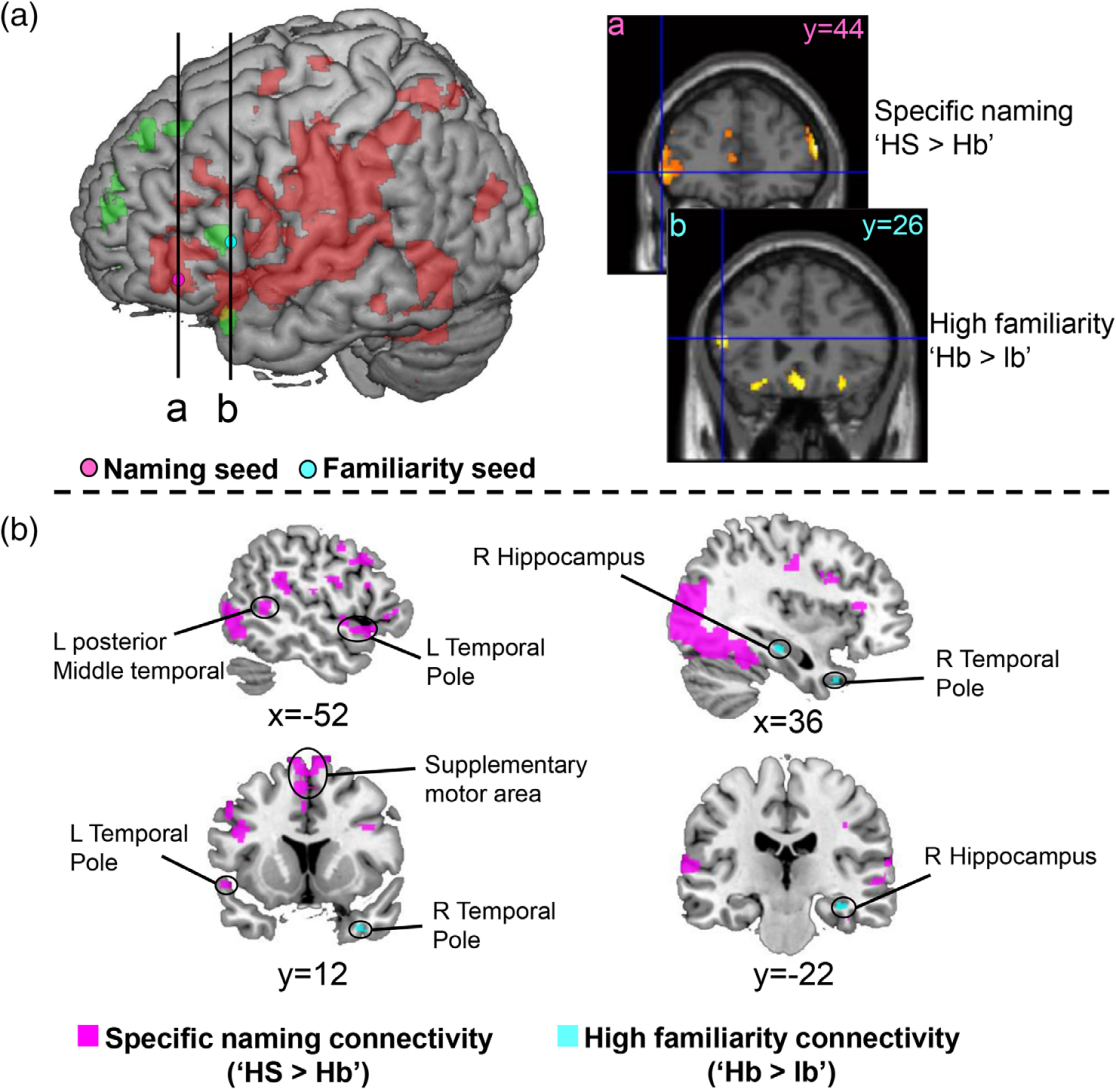
PPI analyses of IFGtri seed areas in contrast: HS > Hb, and contrast: Hb > lb. (a), Seed positions of the naming task PPI analyses. The left part shows the positions of the naming seed (pink) and familiarity seed (cyan) on a 3D brain template. The right part shows the same seeds using coronal slices (i naming seed; ii familiarity seed) to clearly demarcate the anatomical boundaries. (b) Results of the PPI analyses for the two contrasts. Voxels that have significantly stronger connectivity with the naming seed (anterior‐ventral left IFGtri) during the “HS” than the “Hb” condition are shown in pink; voxels that have stronger connectivity with the familiarity seed (posterior‐dorsal left IFGtri) in the “Hb” than the “lb” condition are shown in cyan (*p* < .05, FDR corrected at voxel level). HS, high familiarity under the specific‐naming level; Hb, high familiarity under the basic‐naming level; lb, low familiarity under the basic‐naming level

To examine the functional networks related with the naming effect and familiarity effects, we conducted PPI analysis using two different seeds, which were determined as the peak positions for the contrasts of “HS > Hb” and “Hb > lb” in the left IFGtri (Figure 4a, Table 1), which has been considered as a core region of semantic control particularly related to lexical retrieval (Grabowski et al., 1998; Hurley et al., 2015; Krieger‐Redwood & Jefferies, 2014; Whitney et al., 2011). Figure 4b shows the results of the whole‐brain PPI analysis (the statistical threshold of the group‐level analysis is *p* < .05, FDR corrected at voxel level) of the naming seed (aLIFGtri, −52, 44, −2) and familiarity seed (pLIFGtri, −52 26 14). The naming seed showed increased connectivity in the “HS” condition, compared to the “Hb” condition, with the left TP (BA38), left pMTG (BA21), bilateral STG (BA42), bilateral precentral gyrus (BA6), the left supramarginal gyrus (BA40), and bilateral SMA (BA6) (*p* < .05, FDR corrected at voxel level, Table 2), which are known to be involved in word generation and speech (Hertrich et al., 2016; Indefrey & Levelt, 2004). Conversely, the familiarity seed showed stronger connectivity with the right TP and the right hippocampus in “Hb” than “lb” condition (*p* < .05, FDR corrected at voxel level, Table 2), which have been reported to support recognition of famous objects (e.g., faces of famous persons and famous landmarks) and familiarity feeling (Damasio et al., 2004; Gainotti, 2007; Leveroni et al., 2000; Nakamura et al., 2001). Interestingly, under the specific‐naming contrast (“HS > Hb”), we found that the familiarity seed connected with similar brain regions as results of the naming seed. In contrast, the naming seed did not show significant connectivity with the right TP or hippocampus under high‐familiarity contrast (“Hb > lb”) even when we used liberal threshold (*p* < .005, uncorrected in voxel level). These results suggest that posterior‐dorsal part of the left IFGtri (i.e., familiarity seed) may be involved in the processes related with both the specific naming and the high familiarity effects, while anterior‐ventral part of the left IFGtri (i.e., naming seed) supports only specific naming process. Because we found the significant activations related to either the specific‐naming or familiarity effect in other sub‐region of the left IFG (pars orbitalis, Table 1), we also conducted the PPI analyses using the peak positions in the pars orbitalis for the contrasts of “HS > Hb” and “Hb > lb”. In contrast to the left IFGtri, no significant (*p* < .05, FDR corrected at voxel level) connectivity was observed in the two seeds (−42, 18, −6; −22, 24, −14) in the pars orbitalis of left IFG.

**Table 2:** Brain regions connected with the IFGtri seeds in specific naming, or high familiarity condition

				MNI coordinates		
Brain regions	Left/right	Cluster size (voxels)	*t* value (peak)	*X*	*Y*	Z
**Specific naming (HS > Hb)**						
*Naming seed (−52*, *44*, *−2)*						
Temporal Pole	L	61	3.63	−52	12	−8
Temporal Sup^a^	L	168	5.43	−62	−22	10
Temporal Sup	R	347	5.31	64	−30	16
Frontal Inf Tri^a^	L	253	5.49	−44	32	0
Frontal Inf Orb	R	27	3.23	56	20	−6
Temporal Mid	L	93	5.32	−54	−48	6
Supramarginal^a^	L	90	5.99	−46	−36	26
Supp Motor Area	L/R	903	4.17	−10	6	70
Precentral^a^	L	490	3.86	−44	−6	48
Precentral^a^	R	1,112	5.08	42	−16	38
Parietal Sup	L	56	4.12	−28	−62	54
Fusiform^a^	L	858	4.65	−40	−44	−24
Fusiform^a^	R	845	6.20	32	−46	−14
Occipital Mid^a^	L	1,743	6.60	−36	−88	20
Occipital Mid^a^	R	1,156	5.80	46	−84	4
**High familiarity (Hb > lb)**						
*Familiarity seed (−52*, *26*, *14)*						
Temporal Pole	R	29	4.68	34	12	−36
Hippocampus	R	20	4.15	36	−24	−16

*Note*: Only clusters with a significant activity of voxel‐level threshold *p*_FDR‐corr_ < .05 are reported.

Abbreviations: L, left; R, right; and L/R, the clusters that covered bilateral hemispheres.

a The clusters go through the cluster‐wise FWE correction (*p*_FWE‐corr_ < .05 at cluster level).

One potential question here is whether the anterior‐ventral part of the left IFGtri is recruited only when a participant remembers an object's specific name or the same anterior‐ventral part is recruited whenever a participant remembers semantic knowledge associated with an object voluntarily, including both lexical and nonlexical information. In the former case, the recollection of nonlexical semantic information might entail support from other parts of the left IFGtri for the corresponding modalities such as colors of objects instead.

### 3.4 Connectivity patterns for color and context retrievals

We next investigated how the left IFGtri changed its functional connectivity pattern according to retrieval demands in domains outside of specific‐naming. For this purpose, we measured BOLD signals during the color‐retrieval task and the context‐retrieval task. In these control tasks, the same animal pictures were used as those in the naming task. The participants reported silently whether they retrieved modal‐specific details of the animals by pressing one of the two buttons (left/ “yes,” right/ “no”).

In the control task, as in the connection between the naming levels and the familiarity ratings in the naming task, there was a significant difference in familiarity ratings between the successful and unsuccessful retrieval trials in both the color‐retrieval task [*F* (1, 18) *=* 171.1, *p* < .0001, repeated measures ANOVA] and the context‐retrieval task [*F* (1, 12) *=* 174, *p* < .0001, repeated measures ANOVA]. We found that the percentage of high familiarity rating trials (scores: 5, 6, 7) in unsuccessful retrieval were significantly smaller in both of the two feature‐retrieval tasks (color:17%; context:10.5%) compared with the percentage of basic‐naming (28%) in the naming task (color vs. basic naming: *χ* ^*2*^ *=* 61.1, *p* < .0001; context vs. basic naming: *χ* ^*2*^ *=* 90.5, *p* < .0001). This tendency was stronger in the context‐retrieval task than the color retrieval task (*χ* ^*2*^ *= 17.3*, *p* < .0001). Because of the small number of high‐familiarity rating trials in unsuccessful retrieval, we could not balance these trials with the high‐familiarity rating trials in successful retrieval trials for the feature‐retrieval tasks.

**Figure 5:**
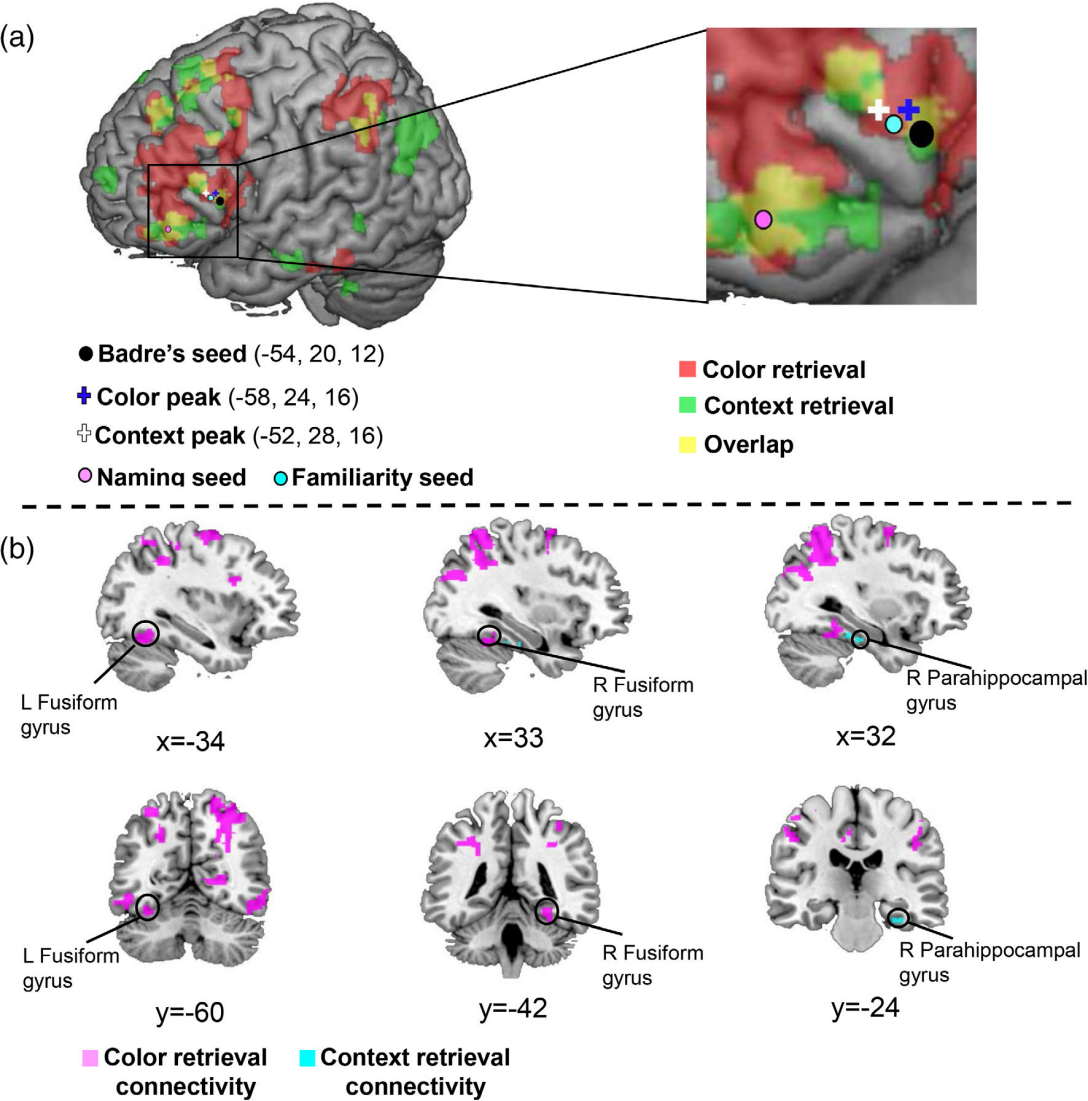
PPI analyses of IFG triangularis seed areas during context retrieval and color retrieval. (a) Seed positions of the feature‐retrieval task PPI analyses. The left part shows the positions of the Badre's seed (black), familiarity seed (cyan), naming seed (pink), and the context peak (white cross), color peak (blue cross) on a 3D brain template. The right part shows their positions in the amplified left IFGtri area. (b) Results of the PPI analyses of Badre's seed. Voxels that have stronger connectivity with this seed during the retrieval of animal‐color information are shown in pink; voxels that have significantly stronger connectivity with this seed during the retrieval of animal‐context information are shown in cyan (*p* < .001, uncorrected at voxel level and *p* < .05, FDR corrected at cluster level)

We examined the activations to the contrasts of “successful retrieval > unsuccessful retrieval” (“Yes > No”) in the color‐retrieval task and the context‐retrieval task separately and found that the left IFGtri showed significant retrieval effects in both tasks (Figure 5a). The activation peaks [(−58, 24, 16) for color‐retrieval; (−52 28 16) for context‐retrieval] were close to the familiarity seed (pLIFGtri, −52, 26, 14) defined by high‐familiarity contrast (“Hb **>** lb”) in the naming task. An important point was that all of these were close to one seed reported in the previous studies [“Badre's seed” (−54, 20, 12), (Badre et al., 2005; Badre & Wagner, 2007; Whitney et al., 2011)] that claimed its pivotal role in cognitive control of semantic memory (Figure 5a). However, compared with our feature‐retrieval tasks asking the participants to recollect modal‐specific contents (i.e., color, context) of individual animals vividly, the previous studies only employed semantic judgment tasks where the participants chose one of the targets based on its semantic relationship with the cue. We conducted PPI analyses using Badre's seed as well as the two seeds determined by the activation peaks for color retrieval and context retrieval, and found that the results using the Badre's seed were consistent with those using the other two seeds (Table 3 and see Table S4; the statistical threshold of the group‐level analysis is *p* < .001, uncorrected at voxel level and *p* < .05, FDR corrected at cluster level). We noted that the Badre's seed (−54, 20, 12) also showed similar results as the familiarity seed (pLIFGtri) in the PPI analyses testing either the specific‐naming contrast or the high‐familiarity contrast. Hereafter, we have demonstrated the results of PPI analyses using the Badre's seed, as the present findings can be directly compared with those of the preceding studies.

**Table 3:** Brain regions connected with the Badre's IFGtri seed in color retrieval or context retrieval task

				MNI coordinates		
Brain regions	Left/right	Cluster size (voxels)	*t* value (peak)	*X*	*Y*	*Z*
*Color retrieval (Y* > *N)*						
Fusiform	L	70	4.44	−32	−60	−18
Fusiform^a^	R	183	5.57	30	−42	−16
Lingual^a^	R	256	5.92	26	−54	2
Frontal Inf Tri^a^	L	91	4.92	−34	10	26
Frontal Inf Tri^a^	R	119	5.86	46	32	24
Supp Motor Area^a^	L	205	5.50	−8	−4	74
Supp Motor Area^a^	R	230	5.06	8	10	60
Precentral^a^	L	221	5.63	−34	−4	62
Frontal Mid^a^	R	139	4.87	30	−2	62
Parietal Sup^a^	L	311	5.42	−22	−66	40
Parietal Sup^a^	R	556	7.24	34	−52	60
Parietal Inf^a^	L	349	5.14	−48	−28	42
*Context retrieval (Y* > *N)*						
Parahippocampal^a^	R	87	8.71	30	−24	−24

*Note*: Only the clusters with a significant activity of voxel‐level *p*_uncorr_ < .001 and cluster‐level *p*_FDR‐corr_ < .05 are reported.

Abbreviations: L, left; R, right.

a The clusters go through the cluster‐wise FWE correction (*p*_FWE‐corr_ < .05).

Figure 5b shows the results of the whole‐brain PPI analysis during the feature‐retrieval tasks. The Badre's seed showed increased connectivity with the bilateral FG, the bilateral SMA, the right lingual gyrus (LG) during the color retrieval (*p* < .001, uncorrected at voxel level and *p* < .05, FDR corrected at cluster level, Table 3). Among these regions, the FG and LG have been implicated in color perception and retrieval of object color knowledge (Hsu, Frankland, & Thompson‐Schill, 2012; Miceli et al., 2001; Simmons et al., 2007; Wang et al., 2013). Conversely, the same seed showed significantly stronger connectivity with the right parahippocampal gyrus (PHC) (*p* < .001, uncorrected at voxel level and *p* < .05, FDR corrected at cluster level, Table 3) during context retrieval. The parahippocampal cortex has been reported to support encoding and retrieving of contextual information (Davachi, 2006; Diana, Yonelinas, & Ranganath, 2007, 2010; Ranganath & Ritchey, 2012; Staresina et al., 2011). These results indicate that the posterior‐dorsal part of left IFGtri connected with different brain regions depending on the modalities of to‐be‐retrieved semantic attributes (i.e., color or context) during the feature‐retrieval task.

### 3.5 Connectivity patterns across multiple semantic attributes of an object

To examine specificity of the connectivity patterns among the modalities (i.e., word, color, and context) of memory contents for their retrieval, we conducted the PPI analyses using the same seeds and the same ROIs in different contrast conditions. In addition to the anterior‐ventral naming seed and the posterior‐dorsal seed (i.e., Badre's seed) in the left IFGtri, we also used a mPFC seed (−14, 60, 18) which showed a significant familiarity (“Hb > lb” contrast) effect in the naming task (Figure 3) as the control. The mPFC has been identified as a key component of episodic memory system (Chao, Huston, Li, Wang, & de Souza Silva, 2016; DeVito & Eichenbaum, 2010; Euston, Gruber, & McNaughton, 2012; Morici, Bekinschtein, & Weisstaub, 2015) particularly for retrieving source information about where events occurred (DeVito & Eichenbaum, 2010). We compared the connectivity patterns between the posterior‐dorsal seed in the left IFGtri and the mPFC seed, both of which showed a significant familiarity effect. In the present study, in total eight ROIs were determined (see Section 2.4.3): the left SMA, left TP, and left pSTG were selected from the specific naming effect; the right TP, and the right hippocampus were from the familiarity effect; the bilateral FG were related to color retrieval; the right parahippocampal gyrus was related to context retrieval (Figures 4 and 5). We calculated the beta values of the ROIs in the PPI analyses testing the four contrasts (specific name, high familiarity, color retrieval, and context retrieval).

**Figure 6:**
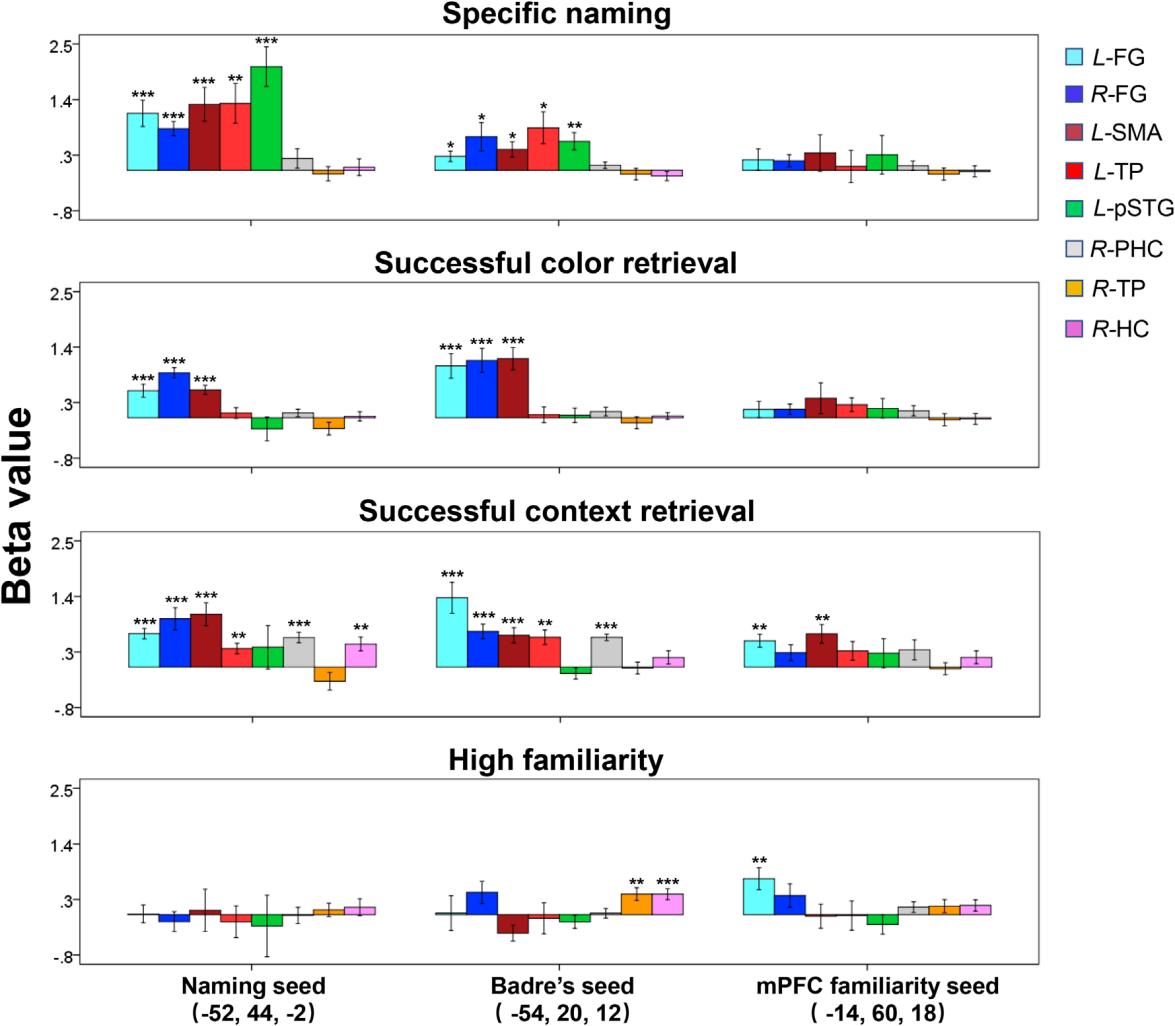
ROI analyses of the PPI effects under different retrieval processes. The bilateral FG and the left SMA showed a significant connectivity with the naming seed (anterior‐ventral left IFGtri, −52, 44, −2) and the Badre's seed (posterior‐dorsal left IFGtri, −54, 20, 12) for all the retrieval conditions including specific name, color and context but not for the familiarity contrast. In addition, the two left IFGtri seeds showed significant connectivity with the left TP, and left pSTG under the specific naming; with the left TP, and right PHC under context retrieval. Compared with the naming seed, the Badre's seed also showed increased connectivity with right TP and HP under the high familiarity condition. In contrast to the two left IFGtri seeds, the mPFC familiarity seed (medial PFC, −14, 60, 18) did not show substantial connectivity under specific naming, and color retrieval. It showed only a small but significant connectivity with the left FG and the left SMA in the context retrieval. The connectivity between mPFC familiarity seed and the left FG was also observed for the high familiarity contrast. Error bars indicate SEM. * *p* < .05 (uncorrected *t*‐test); ** *p* < .05; *** *p* < .01 (Bonferroni corrected *t*‐test). FG, fusiform gyrus; HC, hippocampus; mPFC, medial prefrontal cortex; PHC, parahippocampal gyrus; pSTG, posterior superior temporal gyrus; SMA, supplementary motor area; TP, temporal pole

Figure 6 shows the results of ROI analysis. The bilateral FG and the left SMA showed a significant increase in connectivity with the naming seed (anterior‐ventral part of left IFGtri) and the Badre's seed (posterior‐dorsal part of left IFGtri) for all the retrieval contrasts including name, color and context but not for the familiarity contrast. In addition to these common brain regions, left TP, left pSTG, and right PHC increased their connectivity with the two seeds of the left IFGtri for the specific naming and/or the context retrieval. The patterns of connectivity between the naming seed among the ROIs were similar to those found with the Badre's seed for the all three retrieval‐dependent contrasts. In contrast to the two seeds in the left IFGtri, exhibiting a substantial increase in connectivity in all the three retrieval contrasts, the mPFC familiarity seed showed no such connectivity pattern. It showed only a small but significant increase in connectivity with the left FGtri and the left SMA in the context retrieval. The connectivity between mPFC familiarity seed and the left FG was also observed for the high familiarity contrast. The recruitment of the FG across the seeds as well as across the contrast types may depend on the attributes of the present stimulus set (i.e., visual objects) (Bi, Wang, & Caramazza, 2016). Interestingly, the Badre's seed demonstrated increased connectivity with right TP and HP rather than the left FG under the high familiarity condition.

## 4 DISCUSSION

The present study utilized a novel object naming paradigm to unequivocally distinguish the brain mechanisms associated with specific‐naming and high‐familiarity effects using fMRI. In doing so, we obtained functional‐neuroimaging evidence that may support previous suggestion from neuropsychological studies (Damasio et al., 2004; Drane et al., 2013; Tranel, Damasio, & Damasio, 1997) that the lexical access and semantic recognition stages are differentially processed at the neural level. PPI analyses showed significant connectivity between the anterior‐ventral part of the left IFGtri and brain regions involved in word generation during specific naming as well as between its posterior‐dorsal part and the right TP and hippocampus for the recognition of familiar objects. Furthermore, we detected that the left IFGtri changed its connectivity patterns in accordance with the target modality of semantic retrieval during feature‐retrieval tasks.

The first major finding from the present study is that specific naming and recognition of familiar objects operate via different brain networks. This is the first functional‐neuroimaging evidence that corroborates previous neuropsychological studies showing distinct semantic recognition and lexical retrieval processes in the separate brain regions, as far as we know. In addition to the clear area‐separations in the contrast analysis (Figure 3), the brain areas showing significant connectivity with the left IFGtri differed between the specific‐naming condition and the high‐familiarity condition (Figure 4, Table 2). Under the specific‐naming condition (“HS > Hb”), the anterior‐ventral part of left IFGtri showed stronger connectivity with brain areas, which are reportedly involved in either lexical retrieval (e.g., the left ATL, Damasio et al., 1996, 2004) or speech and word‐production (the STG, supramarginal gyrus, and SMA, Indefrey & Levelt, 2004; Alario, Chainay, Lehericy, & Cohen, 2006; Hickok & Poeppel, 2007; Hickok, 2009; Indefrey, 2011; Hertrich et al., 2016). These results may reflect higher costs for selecting a correct word (i.e., specific naming) among many exemplars of the same semantic category than selecting the category (i.e., basic naming; Howard et al., 2006; Riès, Karzmark, Navarrete, Knight, & Dronkers, 2015; Rosch et al., 1976). The strong connectivity of the left IFGtri to the lexical retrieval and word‐production brain areas under the specific‐naming condition was consistent with previous literature showing the activation of the left IFGtri during an object‐naming task (Grabowski et al., 1998) and the impaired picture naming performance due to the distracted activity of the left IFGtri by the transcranial magnetic stimulation (Krieger‐Redwood & Jefferies, 2014). Together, the present study suggests that the left IFGtri contributes to the lexical processing by interacting with either the lexical retrieval or word‐production brain regions.

Compared with the specific naming, different brain regions have been detected under high‐familiarity condition (“Hb > lb”). In which, the posterior‐dorsal part of left IFGtri connected with the right TP and right hippocampus which have been reported to support recognition and familiarity feeling of familiar objects (Damasio et al., 2004; Gainotti, 2007; Leveroni et al., 2000; Nakamura et al., 2001). Together, a strong contrast has been noted in the left IFGtri between semantic recognition (the posterior‐dorsal part) and specific‐naming retrieval (the anterior‐ventral part) instantiated at the level of functional activation and the spatial pattern of functional connectivity, which suggests a major role for the left IFGtri in both lexical retrieval and semantic recognition.

The present study has detected a significant connectivity of the left IFGtri with the left TP during specific naming, but with the right TP under processing for the familiar objects. These results are of particular interest when compared with the extant literature on the functionality of the ATL in human cognition, for which a number of theories have been proposed. One view holds that the ATL provides the basis for knowledge of unique entities (e.g., famous persons). This theory also suggests a laterality difference between the left and right ATL (Abel et al., 2015; Damasio et al., 1996, 2004; Tranel, 2006; Tranel et al., 1997), wherein the function of lexical associativity (i.e., naming) for unique entities is domain‐specific to the left ATL, and recognition of these items is associated with the right ATL. Nonetheless, another account claims that the ATL supports processing of broader categories of objects (e.g., animal, tool) in addition to processing of unique entities (Lambon Ralph et al., 2017; Patterson, Nestor, & Rogers, 2007; Wong & Gallate, 2012). Supporting evidence for the latter is derived from extensive literature covering convergent neuroscience methods (Pobric, Jefferies, & Lambon Ralph, 2007; Rogers et al., 2006; Shimotake et al., 2015; Visser, Jefferies, Embleton, & Lambon Ralph, 2012). For example, neuropsychological studies of semantic dementia (SD) patients with neural atrophy centering in the ATL have detected object naming deficits for all categories (e.g., animal, tool) at the specific (subordinate) naming level but not at the basic (superordinate) naming level (Hodges et al., 1995; Jefferies & Lambon Ralph, 2006; Lambon Ralph, McClelland, Patterson, Galton, & Hodges, 2001). Our results are consistent with the functional lateralization hypothesis laid out within the first account: the left ATL underlies the naming function, and the right ATL supports familiar object recognition and feeling of familiarity. However, an important prerequisite to these findings is that the present study used images of animal as experimental stimuli. The claim above holds that the ATL only underlies knowledge of unique items (e.g., famous persons, landmarks), excluding generic entities such as animals. On this basis, our results would provide partial support for the “semantic hub” theory; the ATL may indeed serve broader categories of object knowledge. Work to reconcile the functional lateralization hypothesis and “semantic hub” theory may be merited in future studies on the ATL, based on our results and those of the works referenced (Abel et al., 2015; Damasio et al., 1996, 2004; Lambon Ralph et al., 2017; Patterson et al., 2007; Tranel, 2006; Tranel et al., 1997; Wong & Gallate, 2012).

As to the left ATL, there is one methodological concern. In contrast to the other main brain regions shown in the present study, the left temporal pole survived only a liberal threshold (voxel‐wise FDR correction, *p* < .05) (Tables 1 and 2). Previous neuroimaging studies found that the ATL suffers from severe fMRI signal drop‐out (Axelrod & Yovel, 2013; Carr, Rissman, & Wagner, 2010; Olman, Davachi, & Inati, 2009). In the present study, the signal‐to‐fluctuation‐noise‐ratio (Binder et al., 2011; Friedman, Glover, & The FBIRN Consortium, 2006) in the ATL (left, 25.5 ± 2.34; right, 22.5 ± 2.2 across 38 subjects) was only ~ 36% of that in the left IFGtri (65.7 ± 4.94). This signal drop‐out may explain the nonsignificant statistical results in the temporal pole in the relatively strict FWE condition. Together with preceding literature reporting the involvements of the left ATL in object‐naming (Abel et al., 2015; Damasio et al., 1996, 2004; Jefferies & Lambon Ralph, 2006), the present study may suggest an involvement of the left ATL in the specific‐naming condition particularly via an interaction with the semantic control area (i.e., the left IFGtri).

In addition to the left ATL, the present study suggests an involvement of the left pSTG in the specific naming process as a representational brain area. In contrast to the contributions of the left ATL in semantic processing described above, preceding literature suggests a role of the pSTG in phonological processing (Damasio et al., 2004; Indefrey & Levelt, 2004; Graves, Dell, Grabowski, Mehta, & Gordon, 2007; Graves et al., 2008; Hulte'n, Vihla, Laine, & Salmelin, 2009; Indefrey, 2011). One important concern here might be whether or not the left pSTG increased its activity and connectivity to the left IFGtri in the specific‐naming condition because of its definitive or semantic difference from the basic‐naming condition. In other words, can the specific‐naming effect in the pSTG be explained by either the different word frequency in the daily life or the different priming effect during the naming task between the specific (subordinate) and basic (superordinate) names or not? Preceding literature showed that commonly people tended to use basic names of objects rather than the specific names (Rosch et al., 1976). In their naming task, participants used 1,595 times of basic name, but only used 14 times of subordinate name. Previous imaging studies using an object‐naming task reported that the left pSTG increased the activity related to the phonological processing for the low‐frequency words (Graves et al., 2008) as well as showed the repetitive suppression effects (Graves et al., 2007), implying an additional cost for the phonological processing of those words. Together, a reasonable interpretation for the present results might be that the left pSTG showed an increased activation and connectivity with the left IFGtri in the specific‐naming condition because of its higher cost of phonological processing. Although the involvements of the left pSTG may not be directly due to the semantic aspect of specific naming itself, its connectivity to the left IFGtri and the connectivity between the left IFGtri and the left ATL may support the two stage model for the object naming process, which contains the lexical‐retrieval stage and the phonological retrieval stage (see Section 1; Dell, 1986; Dell et al., 1997; Dell & O'Seaghdha, 1992; Foygel & Dell, 2000; Schwartz et al., 2006). The Present study suggests the two stages may interact via the left IFGtri.

The second major finding was that the left IFGtri possesses a task‐dependent connectivity pattern, connecting it with different representational brain regions under corresponding semantic retrieval processes. While involvements of the left IFGtri in the semantic control itself is well attested within the literature (Badre et al., 2005; Krieger‐Redwood & Jefferies, 2014; Novick, Kan, Trueswell, & Thompson‐Schill, 2009; Whitney et al., 2011; Whitney, Kirk, O'Sullivan, Lambon Ralph, & Jefferies, 2012), the pattern of task‐dependent connectivity identified in the present study is a novel discovery. Previous neuropsychological studies of semantic aphasia (SA) patients have indicated that the left IFG, including its pars triangularis part, may not be the storage site for semantic representations but rather the facilitator of top‐down control for the retrieval of semantic information (Corbett, Jefferies, Ehsan, & Lambon Ralph, 2009; Jefferies & Lambon Ralph, 2006). Distinct from the semantic control, semantic representations are assumed to be distributed in or near cortical areas involved in processing corresponding sensory or motor features (Barsalou, 1999; Barsalou, Simmons, Barbey, & Wilson, 2003; Binder & Desai, 2011; Hauk, Johnsrude, & Pulvermuller, 2004; Hsu et al., 2012; Kiefer, Sim, Herrnberger, Grothe, & Hoenig, 2008; Simmons et al., 2007). PPI analyses in the present study showed significant connectivity of the left IFGtri to the bilateral STG, the left supramarginal gyrus during specific naming; to the FG and LG during the color retrieval; to the right parahippocampal gyrus during the context‐retrieval. Together, these results suggest that the left IFGtri may in fact control different modality‐specific representational areas, under correspondingly different semantic retrieval demands.

In addition, several previous studies have suggested that there is a graded functional specialization within the left IFG for the semantic control process (Badre & Wagner, 2007; Lambon Ralph et al., 2017). Commonly, the studies have distinguished two functional subregions in left IFG. The left IFG‐orb controls retrieval process that activates goal‐relevant knowledge (e.g., object color). The left IFG‐tri is involved in postretrieval selection between simultaneously active semantic representations (irrespective of automatically or controlled retrieved). The present study focused on the left IFGtri by examining the connectivity patterns across the sub‐regions as well as the contrast analyses, and showed the functional localization within it along the anterior‐ventral to posterior‐dorsal axis; the anterior‐ventral part of left IFGtri supports specific naming, color retrieval, and context retrieval, while the posterior‐dorsal part of left IFGtri supports processing for familiar objects in addition.

The convergence of networks related to semantic stage including the semantic retrievals onto the left IFGtri might support the lexical retrieval stage, which implies that all three stages (i.e., semantic, lexical retrieval, and phonological stages; see Section 1) may interact in the left IFGtri. Compared with retrieving names, colors, and contexts of items voluntarily, familiarity comes in our mind more automatically. It suggests that the anterior‐ventral part of the left IFGtri correlates with the recollection of task relevant knowledge including specific naming, while posterior‐dorsal part of left IFGtri correlates with executive demands across multiple domains, for example, postretrieval selection (Badre et al., 2005; Badre & Wagner, 2007; Thompson‐Schill et al., 1997) for automatically activated information which would be retrieved from familiar items rather than unfamiliar items.

In summary, our results provide evidence that naming and familiarity effects are embedded in different brain networks. In addition, retrieval for specific names of objects is controlled by the same region in the left IFGtri associated with the retrieval of other attributes (e.g., color). The specific‐naming relevant network includes the classical areas related to word production as well as lexical retrieval. On the other hand, the high‐familiarity relevant network includes the right TP and right hippocampus. Perhaps of foremost importance is our indication that the semantic control functionality of the left IFGtri may be task‐dependent; that is to say, it may connect with different domain‐specific representational brain regions under the corresponding semantic memory retrieval task (i.e., name, color, and context). It would be up to future studies to explore the semantic control hub potentiality of the left IFGtri further in relation to its capacity to handle item knowledge of comprehensive categories, as opposed to strictly unique categories of item (e.g., animals).

## CONFLICT OF INTEREST

The authors declare no competing financial interest.

## Supporting information


**Appendix** **S1.** Supporting Information.

## Data Availability

Availability of DATA The data that support the findings of this study are available from the corresponding author upon reasonable request.
