# Supplementary material for: Convergence of distinct functional networks supporting naming and semantic recognition in the left inferior frontal gyrus
Source: Hum Brain Mapp. 2020 Feb 17;41(9):2389–405. doi: 10.1002/hbm.24953 (PMC7268040; doi:10.1002/hbm.24953)
Supplement: Supplementary file 1 — Appendix S1. Supporting Information. [file HBM-41-2389-s001.docx]

**Supplementary material**

**Supplemental Data Analysis**

*The first type of categorical GLM analysis*

In the first type of categorical GLM analysis of the naming task fMRI data, we conducted two GLMs to examine the naming and familiarity effects separately. As to the naming-level differences, the GLM included 4 main regressors: the effect of specific-naming trials, the effect of basic-naming trials, the effect of baseline condition trials (i.e., scrambled-picture trials), and the effect of other no-interest trials (i.e., the error trials). In addition, the six motion regressors were also included as nuisance regressors. In this GLM, the contrast of interest was “specific-naming > basic-naming”. As to the familiarity-level difference, the GLM included 3 main regressors: the effect of high-familiarity trials, the effect of low familiarity trials, and the effect of baseline condition trials, in addition to the six motion regressors. In this GLM, the contrast of interest was “high-familiarity > low-familiarity”. We put the data during the two runs into the same GLM models with the design matrix consisting of two separable session-specific partitions to calculate the beta value for every interested regressors in each subject. Each partition included the same regressors. The beta values were averaged across the two partitions. After the generation of SPM contrast image for each subject, the contrast images were entered into a group-level random-effects GLM.


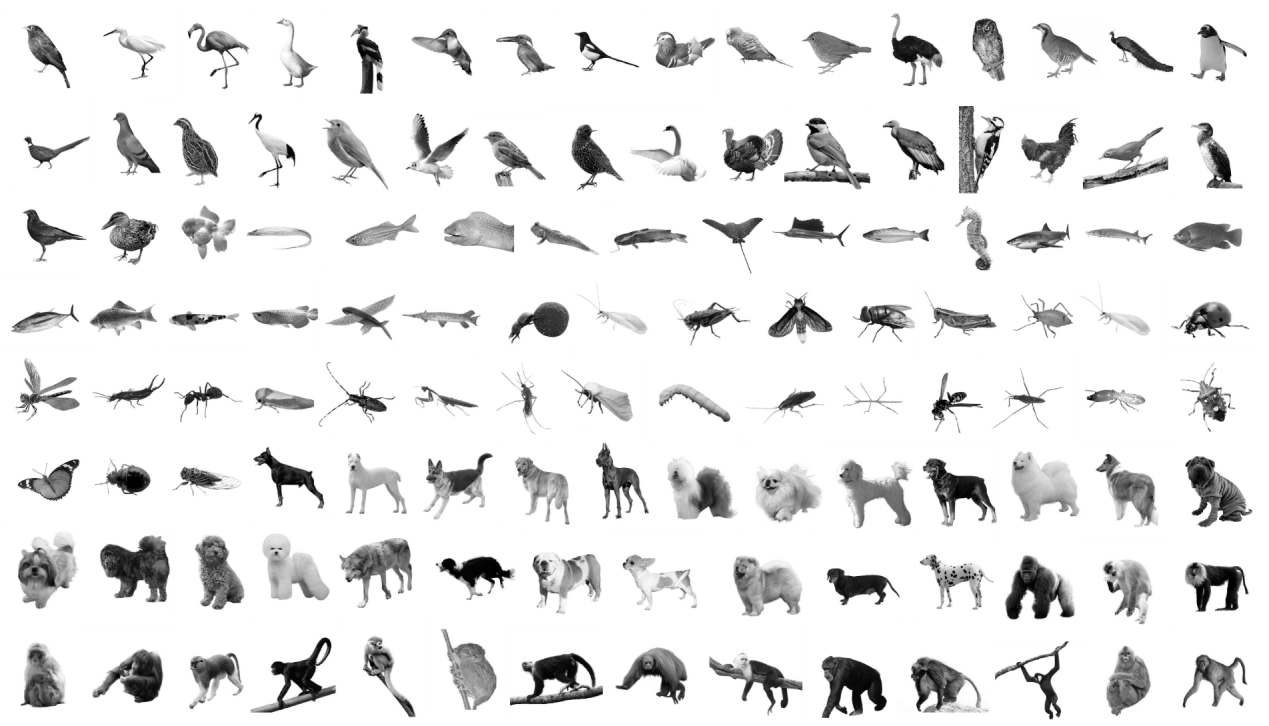


**Supplementary Figure 1. The 120 grayscale animal photographs used in the current study.** The animals included 34 birds, 19 fishes, 27 insects, 23 dogs, and 17 monkeys.


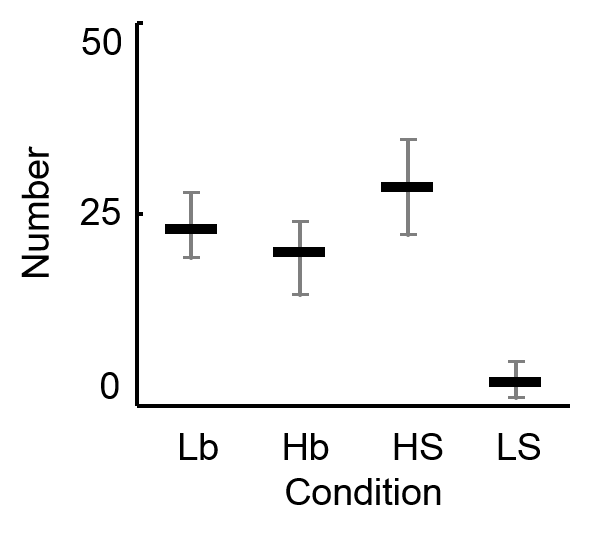


**Supplementary Figure 2.**  Descriptive statistics for trial numbers of per condition (i.e., Lb, Hb, HS and LS). The black thick lines show the medians of per condition. Error bars represent the first quartile and third quartile points. The mean trial numbers across the participants were 29.3±8.3 (“HS”), 20.8 ± 8.1 (“Hb”), 24.3 ±6.9 (“lb” trials), and 3.6±2.9 (“lS”).


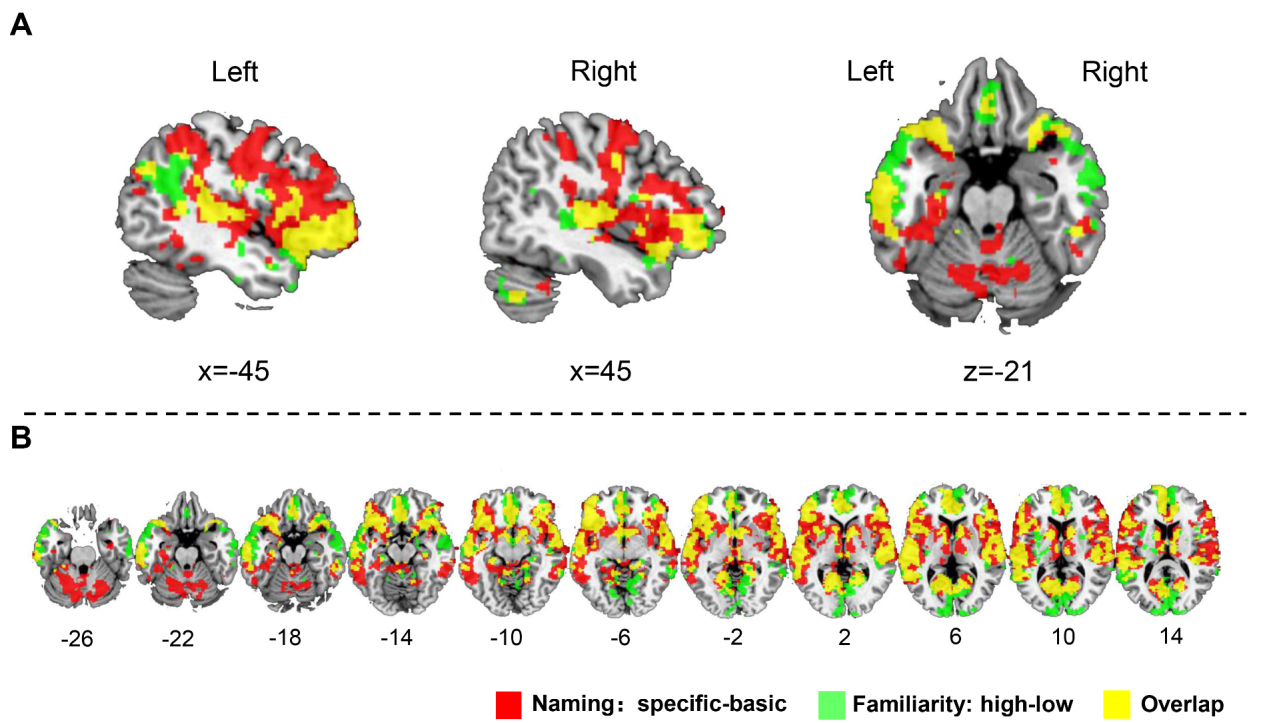


**Supplementary Figure 3. Brain regions associated with specific naming or high familiarity without controlling confounding factors.** ***A,*** Two symmetric sagittal slices and one horizontal slice show high overlapping activations (yellow part) between naming level contrast (red part) and familiarity level contrast (green, *p*＜0.01, FDR corrected at voxel level). ***B,*** Horizontal slices covering the TP and IFG areas.

**Supplementary Table 1.** Head motion parameters of each participant in naming task before correction.

|  | translationX | | translationY | | translationZ | | rotationX | | rotationY | | rotationZ | |
| --- | --- | --- | --- | --- | --- | --- | --- | --- | --- | --- | --- | --- |
| subID | Mean | Max | Mean | Max | Mean | Max | Mean | Max | Mean | Max | Mean | Max |
| sub1 | 0.03 | 0.07 | 0.11 | 0.23 | 0.11 | 0.31 | 0.20 | 0.49 | 0.10 | 0.26 | 0.05 | 0.11 |
| sub2 | 0.03 | 0.09 | 0.03 | 0.12 | 0.08 | 0.30 | 0.06 | 0.22 | 0.05 | 0.16 | 0.06 | 0.14 |
| sub3 | 0.02 | 0.07 | 0.08 | 0.18 | 0.10 | 0.45 | 0.46 | 2.29 | 0.29 | 0.57 | 0.11 | 0.24 |
| sub4 | 0.02 | 0.06 | 0.11 | 0.26 | 0.15 | 0.44 | 0.12 | 0.34 | 0.06 | 0.11 | 0.05 | 0.11 |
| sub5 | 0.04 | 0.12 | 0.04 | 0.18 | 0.11 | 0.36 | 0.18 | 0.48 | 0.18 | 0.60 | 0.08 | 0.21 |
| sub6 | 0.04 | 0.10 | 0.04 | 0.11 | 0.10 | 0.29 | 0.04 | 0.15 | 0.10 | 0.21 | 0.03 | 0.13 |
| sub7 | 0.03 | 0.08 | 0.07 | 0.21 | 0.10 | 0.56 | 0.17 | 0.51 | 0.04 | 0.16 | 0.18 | 0.50 |
| sub8 | 0.03 | 0.07 | 0.21 | 0.36 | 0.10 | 0.82 | 0.29 | 0.80 | 0.07 | 0.18 | 0.04 | 0.08 |
| sub9 | 0.07 | 0.13 | 0.08 | 0.19 | 0.10 | 0.46 | 0.17 | 0.48 | 0.44 | 0.72 | 0.17 | 0.38 |
| sub10 | 0.03 | 0.10 | 0.19 | 0.42 | 0.36 | 0.82 | 0.23 | 0.54 | 0.06 | 0.15 | 0.02 | 0.11 |
| sub11 | 0.03 | 0.07 | 0.15 | 0.35 | 0.25 | 0.58 | 0.11 | 0.30 | 0.08 | 0.26 | 0.07 | 0.19 |
| sub12 | 0.02 | 0.08 | 0.04 | 0.14 | 0.11 | 0.33 | 0.30 | 0.78 | 0.23 | 0.44 | 0.16 | 0.41 |
| sub13 | 0.03 | 0.10 | 0.06 | 0.16 | 0.11 | 0.38 | 0.30 | 0.76 | 0.20 | 0.41 | 0.11 | 0.30 |
| sub14 | 0.04 | 0.13 | 0.09 | 0.26 | 0.21 | 0.65 | 0.16 | 0.42 | 0.09 | 0.21 | 0.05 | 0.19 |
| sub15 | 0.06 | 0.13 | 0.04 | 0.14 | 0.06 | 0.22 | 0.06 | 0.22 | 0.04 | 0.12 | 0.05 | 0.23 |
| sub16 | 0.05 | 0.11 | 0.07 | 0.22 | 0.13 | 0.38 | 0.19 | 0.61 | 0.09 | 0.29 | 0.08 | 0.25 |
| sub17 | 0.03 | 0.10 | 0.06 | 0.16 | 0.06 | 0.26 | 0.16 | 0.45 | 0.13 | 0.49 | 0.02 | 0.09 |
| sub18 | 0.05 | 0.18 | 0.05 | 0.19 | 0.23 | 0.71 | 0.25 | 0.92 | 0.05 | 0.16 | 0.05 | 0.13 |
| sub19 | 0.02 | 0.07 | 0.10 | 0.26 | 0.13 | 0.25 | 0.33 | 0.86 | 0.03 | 0.11 | 0.09 | 0.23 |
| sub20 | 0.03 | 0.15 | 0.06 | 0.17 | 0.11 | 0.43 | 0.12 | 0.33 | 0.07 | 0.25 | 0.04 | 0.15 |
| sub21 | 0.12 | 0.29 | 0.17 | 0.36 | 0.21 | 0.79 | 0.35 | 0.70 | 0.24 | 0.47 | 0.06 | 0.14 |
| sub22 | 0.09 | 0.23 | 0.10 | 0.21 | 0.24 | 0.54 | 0.10 | 0.33 | 0.06 | 0.20 | 0.07 | 0.20 |
| sub23 | 0.05 | 0.11 | 0.08 | 0.25 | 0.16 | 0.44 | 0.11 | 0.32 | 0.15 | 0.34 | 0.18 | 0.42 |
| sub24 | 0.04 | 0.14 | 0.11 | 0.26 | 0.25 | 0.54 | 0.55 | 1.00 | 0.11 | 0.30 | 0.10 | 0.26 |
| sub25 | 0.06 | 0.17 | 0.08 | 0.17 | 0.25 | 0.59 | 0.09 | 0.29 | 0.11 | 0.31 | 0.10 | 0.21 |
| sub26 | 0.03 | 0.09 | 0.06 | 0.17 | 0.14 | 0.64 | 0.06 | 0.23 | 0.19 | 0.38 | 0.07 | 0.16 |
| sub27 | 0.12 | 0.33 | 0.31 | 0.67 | 0.21 | 1.35 | 0.40 | 0.72 | 0.11 | 0.30 | 0.11 | 0.33 |
| sub28 | 0.05 | 0.15 | 0.33 | 0.61 | 0.19 | 0.81 | 0.19 | 0.74 | 0.07 | 0.23 | 0.07 | 0.21 |
| sub29 | 0.03 | 0.06 | 0.09 | 0.22 | 0.18 | 0.44 | 0.15 | 0.56 | 0.07 | 0.22 | 0.04 | 0.12 |
| sub30 | 0.04 | 0.18 | 0.06 | 0.29 | 0.19 | 1.48 | 0.25 | 0.84 | 0.09 | 0.44 | 0.09 | 0.23 |
| sub31 | 0.16 | 0.39 | 0.16 | 0.47 | 0.22 | 0.63 | 0.18 | 0.70 | 0.18 | 0.47 | 0.13 | 0.33 |
| sub32 | 0.04 | 0.15 | 0.04 | 0.14 | 0.28 | 0.78 | 0.12 | 0.39 | 0.19 | 0.45 | 0.10 | 0.43 |
| sub33 | 0.09 | 0.19 | 0.06 | 0.20 | 0.41 | 1.28 | 0.20 | 0.70 | 0.33 | 0.68 | 0.06 | 0.23 |
| sub34 | 0.03 | 0.10 | 0.05 | 0.12 | 0.17 | 0.65 | 0.09 | 0.28 | 0.15 | 0.35 | 0.05 | 0.13 |
| sub35 | 0.04 | 0.13 | 0.10 | 0.20 | 0.19 | 0.53 | 0.35 | 0.92 | 0.26 | 0.59 | 0.10 | 0.20 |
| sub36 | 0.04 | 0.10 | 0.09 | 0.24 | 0.36 | 1.00 | 0.26 | 0.69 | 0.06 | 0.19 | 0.04 | 0.15 |
| sub37 | 0.14 | 0.31 | 0.68 | 1.13 | 0.21 | 0.48 | 0.31 | 1.13 | 0.09 | 0.30 | 0.15 | 0.33 |
| sub38 | 0.06 | 0.17 | 0.04 | 0.13 | 0.23 | 0.82 | 0.15 | 0.38 | 0.15 | 0.36 | 0.09 | 0.14 |

Unit of translation is millimeter; unit of rotation is degree.

**Supplementary Table 2.** Head motion parameters of each participant in naming task after correction.

|  | translationX | | translationY | | translationZ | | rotationX | | rotationY | | rotationZ | |
| --- | --- | --- | --- | --- | --- | --- | --- | --- | --- | --- | --- | --- |
| subID | Mean | Max | Mean | Max | Mean | Max | Mean | Max | Mean | Max | Mean | Max |
| sub1 | 0.0002 | 0.0007 | 0.0011 | 0.0030 | 0.0013 | 0.0036 | 0.0012 | 0.0037 | 0.0008 | 0.0039 | 0.0003 | 0.0014 |
| sub2 | 0.0002 | 0.0005 | 0.0005 | 0.0012 | 0.0011 | 0.0040 | 0.0014 | 0.0047 | 0.0007 | 0.0026 | 0.0002 | 0.0010 |
| sub3 | 0.0010 | 0.0031 | 0.0010 | 0.0033 | 0.0026 | 0.0098 | 0.0022 | 0.0117 | 0.0022 | 0.0061 | 0.0012 | 0.0031 |
| sub4 | 0.0009 | 0.0027 | 0.0021 | 0.0058 | 0.0069 | 0.0177 | 0.0040 | 0.0111 | 0.0015 | 0.0065 | 0.0019 | 0.0052 |
| sub5 | 0.0012 | 0.0034 | 0.0008 | 0.0029 | 0.0056 | 0.0130 | 0.0072 | 0.0146 | 0.0041 | 0.0119 | 0.0012 | 0.0041 |
| sub6 | 0.0004 | 0.0011 | 0.0002 | 0.0006 | 0.0012 | 0.0037 | 0.0012 | 0.0040 | 0.0014 | 0.0034 | 0.0003 | 0.0010 |
| sub7 | 0.0009 | 0.0030 | 0.0007 | 0.0024 | 0.0019 | 0.0070 | 0.0019 | 0.0058 | 0.0008 | 0.0033 | 0.0014 | 0.0047 |
| sub8 | 0.0003 | 0.0019 | 0.0007 | 0.0029 | 0.0024 | 0.0062 | 0.0012 | 0.0055 | 0.0006 | 0.0045 | 0.0003 | 0.0012 |
| sub9 | 0.0020 | 0.0047 | 0.0013 | 0.0043 | 0.0012 | 0.0044 | 0.0017 | 0.0055 | 0.0016 | 0.0041 | 0.0006 | 0.0034 |
| sub10 | 0.0005 | 0.0020 | 0.0004 | 0.0023 | 0.0023 | 0.0076 | 0.0046 | 0.0118 | 0.0007 | 0.0031 | 0.0005 | 0.0022 |
| sub11 | 0.0009 | 0.0030 | 0.0007 | 0.0021 | 0.0032 | 0.0131 | 0.0034 | 0.0107 | 0.0009 | 0.0030 | 0.0003 | 0.0013 |
| sub12 | 0.0013 | 0.0027 | 0.0011 | 0.0036 | 0.0015 | 0.0042 | 0.0012 | 0.0035 | 0.0009 | 0.0030 | 0.0005 | 0.0016 |
| sub13 | 0.0007 | 0.0024 | 0.0006 | 0.0028 | 0.0025 | 0.0068 | 0.0030 | 0.0096 | 0.0014 | 0.0047 | 0.0012 | 0.0030 |
| sub14 | 0.0006 | 0.0017 | 0.0006 | 0.0027 | 0.0012 | 0.0081 | 0.0015 | 0.0058 | 0.0011 | 0.0048 | 0.0005 | 0.0030 |
| sub15 | 0.0007 | 0.0020 | 0.0006 | 0.0025 | 0.0010 | 0.0041 | 0.0031 | 0.0071 | 0.0009 | 0.0035 | 0.0009 | 0.0025 |
| sub16 | 0.0004 | 0.0013 | 0.0011 | 0.0036 | 0.0017 | 0.0062 | 0.0048 | 0.0153 | 0.0019 | 0.0064 | 0.0006 | 0.0029 |
| sub17 | 0.0005 | 0.0023 | 0.0006 | 0.0026 | 0.0029 | 0.0080 | 0.0013 | 0.0055 | 0.0013 | 0.0040 | 0.0006 | 0.0021 |
| sub18 | 0.0008 | 0.0047 | 0.0016 | 0.0047 | 0.0040 | 0.0158 | 0.0070 | 0.0241 | 0.0012 | 0.0040 | 0.0026 | 0.0087 |
| sub19 | 0.0003 | 0.0012 | 0.0006 | 0.0023 | 0.0017 | 0.0067 | 0.0072 | 0.0185 | 0.0026 | 0.0084 | 0.0019 | 0.0049 |
| sub20 | 0.0008 | 0.0026 | 0.0006 | 0.0019 | 0.0014 | 0.0047 | 0.0034 | 0.0090 | 0.0013 | 0.0051 | 0.0006 | 0.0026 |
| sub21 | 0.0010 | 0.0031 | 0.0037 | 0.0201 | 0.0053 | 0.0107 | 0.0075 | 0.0317 | 0.0035 | 0.0094 | 0.0014 | 0.0036 |
| sub22 | 0.0012 | 0.0035 | 0.0008 | 0.0024 | 0.0035 | 0.0106 | 0.0010 | 0.0037 | 0.0012 | 0.0039 | 0.0008 | 0.0030 |
| sub23 | 0.0013 | 0.0052 | 0.0019 | 0.0062 | 0.0020 | 0.0063 | 0.0032 | 0.0100 | 0.0019 | 0.0059 | 0.0015 | 0.0047 |
| sub24 | 0.0008 | 0.0032 | 0.0013 | 0.0038 | 0.0040 | 0.0078 | 0.0064 | 0.0149 | 0.0030 | 0.0071 | 0.0006 | 0.0019 |
| sub25 | 0.0011 | 0.0037 | 0.0005 | 0.0040 | 0.0014 | 0.0094 | 0.0015 | 0.0056 | 0.0008 | 0.0029 | 0.0014 | 0.0029 |
| sub26 | 0.0005 | 0.0016 | 0.0010 | 0.0033 | 0.0012 | 0.0054 | 0.0014 | 0.0052 | 0.0021 | 0.0060 | 0.0010 | 0.0030 |
| sub27 | 0.0019 | 0.0060 | 0.0022 | 0.0047 | 0.0049 | 0.0118 | 0.0056 | 0.0134 | 0.0019 | 0.0059 | 0.0005 | 0.0023 |
| sub28 | 0.0008 | 0.0035 | 0.0012 | 0.0045 | 0.0025 | 0.0094 | 0.0034 | 0.0113 | 0.0018 | 0.0047 | 0.0004 | 0.0021 |
| sub29 | 0.0002 | 0.0005 | 0.0006 | 0.0022 | 0.0018 | 0.0042 | 0.0027 | 0.0105 | 0.0007 | 0.0029 | 0.0005 | 0.0021 |
| sub30 | 0.0011 | 0.0037 | 0.0015 | 0.0066 | 0.0023 | 0.0283 | 0.0030 | 0.0145 | 0.0022 | 0.0073 | 0.0007 | 0.0044 |
| sub31 | 0.0017 | 0.0050 | 0.0014 | 0.0053 | 0.0028 | 0.0095 | 0.0025 | 0.0071 | 0.0012 | 0.0042 | 0.0011 | 0.0049 |
| sub32 | 0.0010 | 0.0044 | 0.0007 | 0.0025 | 0.0019 | 0.0062 | 0.0010 | 0.0041 | 0.0009 | 0.0041 | 0.0005 | 0.0021 |
| sub33 | 0.0018 | 0.0062 | 0.0007 | 0.0027 | 0.0026 | 0.0069 | 0.0046 | 0.0106 | 0.0010 | 0.0044 | 0.0013 | 0.0032 |
| sub34 | 0.0008 | 0.0036 | 0.0011 | 0.0031 | 0.0030 | 0.0115 | 0.0026 | 0.0079 | 0.0024 | 0.0079 | 0.0003 | 0.0015 |
| sub35 | 0.0011 | 0.0034 | 0.0014 | 0.0039 | 0.0027 | 0.0088 | 0.0023 | 0.0075 | 0.0022 | 0.0052 | 0.0006 | 0.0031 |
| sub36 | 0.0008 | 0.0027 | 0.0009 | 0.0040 | 0.0008 | 0.0050 | 0.0050 | 0.0246 | 0.0008 | 0.0033 | 0.0007 | 0.0030 |
| sub37 | 0.0004 | 0.0011 | 0.0047 | 0.0089 | 0.0047 | 0.0098 | 0.0047 | 0.0117 | 0.0011 | 0.0042 | 0.0016 | 0.0039 |
| sub38 | 0.0006 | 0.0016 | 0.0008 | 0.0027 | 0.0010 | 0.0072 | 0.0010 | 0.0056 | 0.0018 | 0.0053 | 0.0003 | 0.0013 |

Unit of translation is millimeter; unit of rotation is degree.

**Supplementary Table 3.** Brain regions were parametrically modulated by the familiarity ratings.

|  |  |  |  | MNI coordinates | | |
| --- | --- | --- | --- | --- | --- | --- |
| Brain Regions | Left/Right | Cluster Size (Voxels) | *t* Value  (peak) | *X* | *Y* | *Z* |
| Temporal pole | L | 53 | 5.17 | -34 | 18 | -26 |
|  | R | 10 | 3.74 | 56 | 14 | -10 |
| Hippocampus | R | 24 | 4.69 | 20 | -6 | -14 |
| Rectus | L/R | 187 | 5.16 | -2 | 36 | -18 |
| Frontal Med Orb | L/R | 334 | 5.24 | -2 | 48 | -12 |
| Frontal Sup Medial | L/R | 804 | 5.38 | 0 | 56 | 18 |
|  | R | 65 | 4.00 | 62 | -4 | -18 |
| Insula | L | 163 | 6.19 | -30 | 20 | -18 |
| Frontal Inf Tri | L | 396 | 5.62 | -48 | 36 | 0 |
| Frontal Inf Orb | L | 380 | 4.90 | -40 | 28 | -12 |
|  | R | 278 | 4.81 | 42 | 32 | -10 |
| Frontal Mid | L | 72 | 4.04 | -34 | 52 | 16 |
|  | R | 21 | 4.40 | 48 | 22 | 48 |
| Cingulum Ant | L | 22 | 5.09 | -2 | 48 | 10 |
| Supp Motor Area | L | 91 | 4.66 | -8 | 22 | 56 |
| Precuneus | L | 93 | 4.12 | -2 | -56 | 24 |
| Lingual | L | 58 | 4.46 | -16 | -48 | -2 |
|  | R | 48 | 4.73 | 18 | -44 | 0 |
| Calcarine | L | 82 | 5.15 | -16 | -54 | 4 |
|  | R | 62 | 4.25 | 10 | -72 | 4 |
| Occipital Sup | L | 180 | 5.21 | -12 | -96 | 6 |

Only clusters with a significant activity of voxel-level threshold *p_FDR-corr_*＜0.05 are reported. L, left; R, right; and L/R, the clusters that covered bilateral hemispheres

**Supplementary Table 4.** Brain regions connected with the IFGtri seeds in color retrieval, or context retrieval task.

|  |  |  |  | MNI Coordinates | | |
| --- | --- | --- | --- | --- | --- | --- |
| Brain Regions | Left/Right | Cluster Size (Voxels) | *t* Value  (peak) | *X* | *Y* | *Z* |
| **Color Retrieval (Y＞N)** |  |  |  |  |  |  |
| *Color Seed (-58, 24, 16)* | | | | | | |
| Fusiform | L | 228 | 5.48 | -34 | -50 | -16 |
|  | R | 284 | 5.33 | 40 | -54 | -22 |
| Lingual | R | 60 | 5.22 | 26 | -54 | 2 |
| Supp Motor Area | L | 104 | 5.28 | -6 | -2 | 74 |
|  | R | 62 | 4.89 | 6 | 10 | 62 |
| Precentral | L | 240 | 5.53 | -32 | -10 | 60 |
| Parietal Sup | L | 93 | 4.29 | -26 | -62 | 58 |
|  | R | 211 | 6.36 | 24 | -60 | 66 |
| Parietal Inf | L | 49 | 4.98 | -50 | -38 | 56 |
| **Context Retrieval (Y＞N)** |  |  |  |  |  |  |
| *Context Seed (-52, 28, 16)* | | | | | | |
| Parahippocampal | R | 83 | 5.97 | 36 | -20 | -22 |
| Supp Motor Area | L/R | 401 | 8.06 | -12 | -4 | 56 |
| Parietal Inf | L | 77 | 5.90 | -32 | -50 | 54 |
| Fusiform | L | 179 | 7.23 | -32 | -38 | -22 |
| Occipital Inf | L | 181 | 6.02 | -30 | -86 | -12 |
|  | R | 373 | 7.51 | 36 | -76 | -4 |

Only clusters with a significant activity of voxel-level *p_uncorr_*＜0.001 and cluster-level *p_FDR-corr_*＜0.05 are reported. L, left; R, right; and L/R, the clusters that covered bilateral hemispheres
